# Ku Must Load Directly onto the Chromosome End in Order to Mediate Its Telomeric Functions

**DOI:** 10.1371/journal.pgen.1002233

**Published:** 2011-08-11

**Authors:** Christopher R. Lopez, Albert Ribes-Zamora, Sandra M. Indiviglio, Christopher L. Williams, Svasti Haricharan, Alison A. Bertuch

**Affiliations:** 1 Department of Pediatrics, Baylor College of Medicine, Houston, Texas, United States of America; 2 Cell and Molecular Biology Interdepartmental Program, Baylor College of Medicine, Houston, Texas, United States of America; 3 Department of Molecular and Cellular Biology, Baylor College of Medicine, Houston, Texas, United States of America; National Institute of Diabetes and Digestive and Kidney Diseases, United States of America

## Abstract

The Ku heterodimer associates with the *Saccharomyces cerevisiae* telomere, where it impacts several aspects of telomere structure and function. Although Ku avidly binds DNA ends via a preformed channel, its ability to associate with telomeres via this mechanism could be challenged by factors known to bind directly to the chromosome terminus. This has led to uncertainty as to whether Ku itself binds directly to telomeric ends and whether end association is crucial for Ku's telomeric functions. To address these questions, we constructed DNA end binding–defective Ku heterodimers by altering amino acid residues in Ku70 and Ku80 that were predicted to contact DNA. These mutants continued to associate with their known telomere-related partners, such as Sir4, a factor required for telomeric silencing, and TLC1, the RNA component of telomerase. Despite these interactions, we found that the Ku mutants had markedly reduced association with telomeric chromatin and null-like deficiencies for telomere end protection, length regulation, and silencing functions. In contrast to Ku null strains, the DNA end binding defective Ku mutants resulted in increased, rather than markedly decreased, imprecise end-joining proficiency at an induced double-strand break. This result further supports that it was the specific loss of Ku's telomere end binding that resulted in telomeric defects rather than global loss of Ku's functions. The extensive telomere defects observed in these mutants lead us to propose that Ku is an integral component of the terminal telomeric cap, where it promotes a specific architecture that is central to telomere function and maintenance.

## Author Summary

The telomeric cap modulates telomere replication and prevents natural chromosome ends from being processed as DNA double-strand breaks (DSBs). In multiple species, including budding yeast, a detailed picture exists of the factors that comprise the telomeric cap and how they associate with telomeric DNA. It is less clear where to place Ku, a conserved heterodimer involved in multiple aspects of telomere biology and DSB repair. Although Ku avidly binds DNA ends, its access to telomeric ends might be restricted by telomere binding proteins and/or higher-order telomere structure. Ku might also be recruited to telomeres via its telomere-associated binding partners. Here, we address whether Ku loads directly onto telomeric ends and whether direct DNA binding is crucial for its telomeric functions. Using structure-guided mutagenesis, we generated end binding–defective yeast Ku heterodimers that retained the ability to associate with Ku's known telomeric binding partners. These end binding–defective heterodimers showed a dramatic reduction in telomere association and were defective for all of Ku's telomeric functions. Our findings indicate that Ku is indeed a component of the telomere cap and that its loading onto telomeric ends is crucial for its telomeric functions and, perhaps, a specific telomere architecture.

## Introduction

Eukaryotic telomeres are comprised of repetitive G-rich sequence arrays and a host of proteins that associate with the repeats directly or as part of a complex [1], [2]. These proteins maintain telomeres by facilitating the replication of telomeric DNA and by protecting the natural chromosomal ends from end-joining and resection activities associated with the repair of DNA double-strand breaks (DSBs). Perturbation of the normal structure and function of the telomere, through the loss of telomere sequence or telomere-associated proteins, can result in genomic instability, checkpoint activation, and cellular senescence or apoptosis [3].

Understanding the structural relationship of telomere-associated proteins with telomeric DNA is integral to our comprehension of the maintenance, structure, and function of telomeres. To date, proteins have been shown to associate with telomeric DNA via at least one of three mechanisms. Some telomere-associated proteins, such as Rap1 in *Saccharomyces cerevisiae* [4] and TRF1 and TRF2 in mammalian cells [5], utilize a myb/homeodomain to bind directly and with high affinity to double-stranded (ds) telomeric DNA sequences. Other proteins, such as Cdc13 in *S. cerevisiae* [6] and the TEBPα/β heterodimer in *Oxytricha nova* [7], utilize one or more oligonucleotide-oligosaccharide binding (OB) folds to associate avidly and specifically with single-stranded (ss) G-rich telomeric DNA. Because telomeres terminate with G-rich overhangs, ss telomeric DNA binding proteins are localized to the chromosome end. The third mechanism by which proteins associate with telomeres is via protein-protein interactions. For example, in *S. cerevisiae*, Sir3 and Sir4 are recruited to telomeric repeat-containing chromatin via Rap1 [8], and in mammalian cells, TIN2 localizes to ds telomeric repeats via its interaction with TRF1 and TRF2 [9], [10]. Proteins capable of binding telomeric DNA directly also can employ this mode of association. For example, POT1, a protein that binds directly to the G-overhang through its OB folds [11], also associates with ds telomeric repeats through a series of protein-protein interactions terminating with TRF1 [12]. Thus, the ability to bind to telomeric DNA directly does not preclude telomeric association via protein-protein interactions.

In contrast to the above examples, the mechanism for the evolutionarily conserved Ku heterodimer's association to the telomere is uncertain. Comprised of the Ku70 and Ku80 subunits, Ku loads onto DNA ends via a preformed ring consisting of an expansive base and narrow bridge that encircles the DNA [13], [14]. Ku binds DNA ends in a sequence-independent manner through a limited number of contacts with the sugar phosphate backbone [14]. Even though Ku binds DNA in a sequence independent manner, it does load onto DNA ends in a specific orientation. The loading face is comprised predominantly of Ku80, while the lagging face consists mostly of Ku70. This orientation is possibly dictated by steric and electrostatic features present on the lagging face that impede the DNA's access to this end of the DNA binding channel.

Given its high affinity for DNA ends, Ku could localize to telomeric chromatin through direct DNA end binding [15]. *In vitro* assays have shown that Ku does associate with a telomeric DNA substrate in this way, as long as it is allowed access to the DNA before Cdc13 is added [16]. However, Cdc13 is associated with telomeres throughout the cell cycle *in vivo* [17]. Thus, the means by which telomeric end binding by Ku might occur *in vivo* in the context of Cdc13 and other telomeric binding proteins that avidly bind either ds or ss telomeric DNA remains unclear. In addition, the telomeres of many species are thought to exist in a higher order structure known as a t-loop, where the terminal G-strand 3′ overhang invades proximal ds telomeric DNA to form a d-loop, thereby concealing the DNA end [18]. In fact, one proposed function of this structure is to prevent Ku's association with the chromosome's terminus, in turn, preventing telomeres from engaging in Ku-dependent, nonhomologous end-joining (NHEJ) [19].

In addition to binding DNA, Ku70 and Ku80 each have N terminal α/β domains which lie laterally to the DNA binding channel and are thought to mediate Ku's interaction with other factors [14]. Ku has been shown to bind or co-purify with telomere-associated factors such as Sir4 in budding yeast and RAP1, TRF1, and TRF2 in mammalian cells [20]–[23]. In the case of Sir4, this has been shown to require residues in the yeast Ku80 α/β domain. Also, Ku's interaction with TLC1, the RNA component of telomerase, is disrupted by a mutation (*yku80-135i*) that maps to the Ku80 α/β domain [24]. Therefore, Ku might also associate with telomeres via its interaction with telomeric factors. Consistent with this, the association of budding yeast Ku80 with subtelomeric chromatin is reduced in the absence of Sir4 [25]. Thus, the possibility exists that Ku is recruited to telomeric repeats through these interactions, independent of its DNA end binding activity.

Defining how Ku associates with telomeres is crucial for understanding its non-overlapping roles in a myriad of telomeric processes. For example, in *S. cerevisiae*, Ku is required for the localization of Est2, the catalytic subunit of yeast telomerase, to telomeres in G1 [17]. This recruitment depends upon a direct interaction between Ku and a 48-nt stem-loop in TLC1 [24]. Ku also protects the telomeric 5′ strand from resection by Exo1 [26], [27], a 5′-3′ exonuclease and flap-endonuclease involved in a variety of DNA repair processes including resection of DSBs [28], [29]. Studies with the Yku80 separation-of-function mutant *yku80-135i*, however, indicate that Ku can regulate telomere length independently of its end protection function [24].

In addition to, and distinct from, its role in telomere replication, Ku aids in the formation of silent telomeric chromatin [30], which results in transcriptional repression of adjacent genes, a phenomenon referred to as *telomeric silencing* or *telomere position effect* [31]. Ku promotes the recruitment of Sir4 to sub-telomeric DNA, [25], [32], [33] and separation-of-function mutants have demonstrated that Ku's interaction with Sir4, while required for Ku's role in telomeric silencing, is dispensable for Ku's role in telomere end protection and length homeostasis [34].

Yeast Ku also is required for the normal localization of telomeres at the nuclear periphery via two pathways that are independent of Sir4 and silent chromatin [35], [36]. One of these pathways is S-phase specific and relies on Ku's interaction with TLC1 and telomerase's interaction with the integral nuclear membrane protein Mps3 [37]. Although perinuclear tethering can promote telomeric silencing, Ku's influence on silencing can occur, at least at some telomeres, independently of its effects on tethering. This is supported by the finding that the perinuclear localization of truncated versions of Tel VI-R and Tel VII-L is maintained in a *yku70-Δ* background (i.e., the tethering of these telomeres is Ku-independent), whereas the telomeric silencing of Tel VI-R *ADE2* and Tel VII-L *URA3* reporters is lost (i.e., the silencing of these telomeres is Ku-dependent) [35], [38].

In addition to telomeres, Ku localizes to DSBs, where it plays a crucial role in NHEJ. Separation-of-function mutations that result in isolated NHEJ or telomeric defects suggest that Ku plays distinct roles at telomeric *versus* broken ends [20], [24], [34], [36], [39], [40]. For example, mutations in helix 5 of the yeast Ku70 α/β domain impact selectively on NHEJ, whereas mutations in helix 5 of the yeast Ku80 α/β domain impact selectively on telomeric silencing [40]. The structural position of NHEJ *versus* telomeric function-specific residues and the polarity with which the heterodimer loads onto DNA ends led us to propose a ‘two-face’ model for how Ku's NHEJ and telomeric functions are spatially organized [40]. In this model, we proposed that Ku's outward face, juxtaposed with the DNA terminus when bound to a broken end, is dedicated to its NHEJ functions, whereas its inward face, which would be oriented toward telomeric chromatin when bound to a telomeric end, is dedicated to Ku's telomeric functions. This model assumes, however, that Ku loads directly onto telomeric ends and that Ku's telomeric functions are dependent upon this mode of binding.

To determine whether Ku associates with telomeres and mediates one or more of its telomeric roles via end binding, we generated DNA end binding defective *S. cerevisiae* Ku heterodimers and examined their association with telomeric chromatin and their impact on telomere function. We found that the loss of DNA end binding activity greatly reduced Ku's association with telomeres and rendered Ku deficient in its ability to protect telomeres from nucleolytic processing, to maintain telomere length, and to contribute to the formation of silent telomeric chromatin. Therefore, Ku must access and directly associate with telomeric ends in order for telomeres to adopt their proper architecture and perform their genome-protective functions.

## Results

### Construction of DNA end binding–defective Ku heterodimers

**Figure 1:**
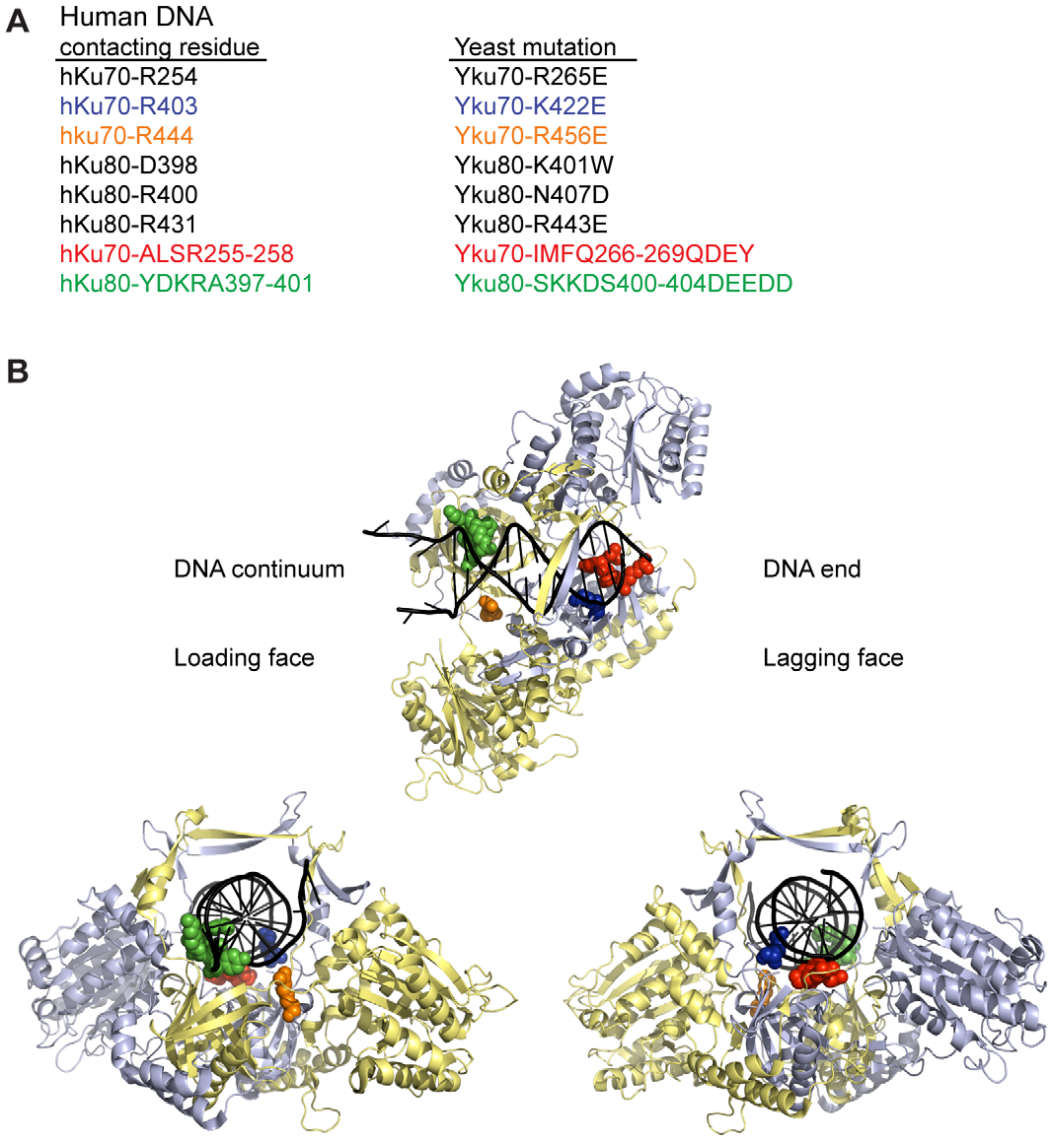
Positions of human Ku DNA contacting residues targeted in yeast Ku. (A) Selected DNA contacting residues in human Ku and the aligning residues in yeast, with the specified amino acid substitutions created by mutagenesis. The structural positions of the subset highlighted in blue, orange, red, and green are shown in B. (B) Structure of human Ku bound to DNA [14] with a subset of residues, corresponding by color to those listed in A, represented as spheres. The remainder of Ku70 is colored in pale blue, Ku80 in yellow, and DNA in black. The top representation is a top view of Ku demonstrating the expansive base and narrow bridge forming the DNA binding channel. Note that the residues corresponding to those in the Yku80 panel mutation and Yku70-R456E map to the loading face, whereas the residues corresponding to those in the Yku70 panel mutation and Yku70-K422 map to the lagging face.

To determine whether DNA end binding is required for Ku's association with telomeres and its telomeric functions, we sought to create *S. cerevisiae* Ku heterodimers that were specifically defective in their ability to bind DNA ends by mutagenizing residues that interact with DNA. We used the crystal structure of human Ku bound to DNA (PDB 1JEY) [14] and multiple sequence alignments containing yeast and human Ku70 (Yku70 or hKu70) and Ku80 (Yku80 or hKu80) subunits to identify potential DNA contacting residues in yeast Ku. Twenty-four residues in human Ku, distributed between the two subunits, contact DNA in the crystal structure. Because of poor sequence conservation between most of these residues and the aligning residues in yeast, we limited single amino acid substitutions to a subset of five residues that ranked in the top 20th percentile of our previous analyses of evolutionarily conserved amino acid residues of Ku70 and Ku80 [40] (Figure 1). We also targeted Yku70-R456, the yeast residue that aligned with hKu70-R444, which was in close proximity to DNA in the human Ku-DNA co-crystal and highly conserved throughout evolution. In addition to these single amino acid substitutions, we targeted residues that corresponded to small runs of DNA contacting residues in each of the human subunits (Yku70-IMFQ266-269 and Yku80-SKKDS400-404 which correspond to hKu70-ALSR255-258 and hKu80-YDKRA397-401).

**Figure 2:**
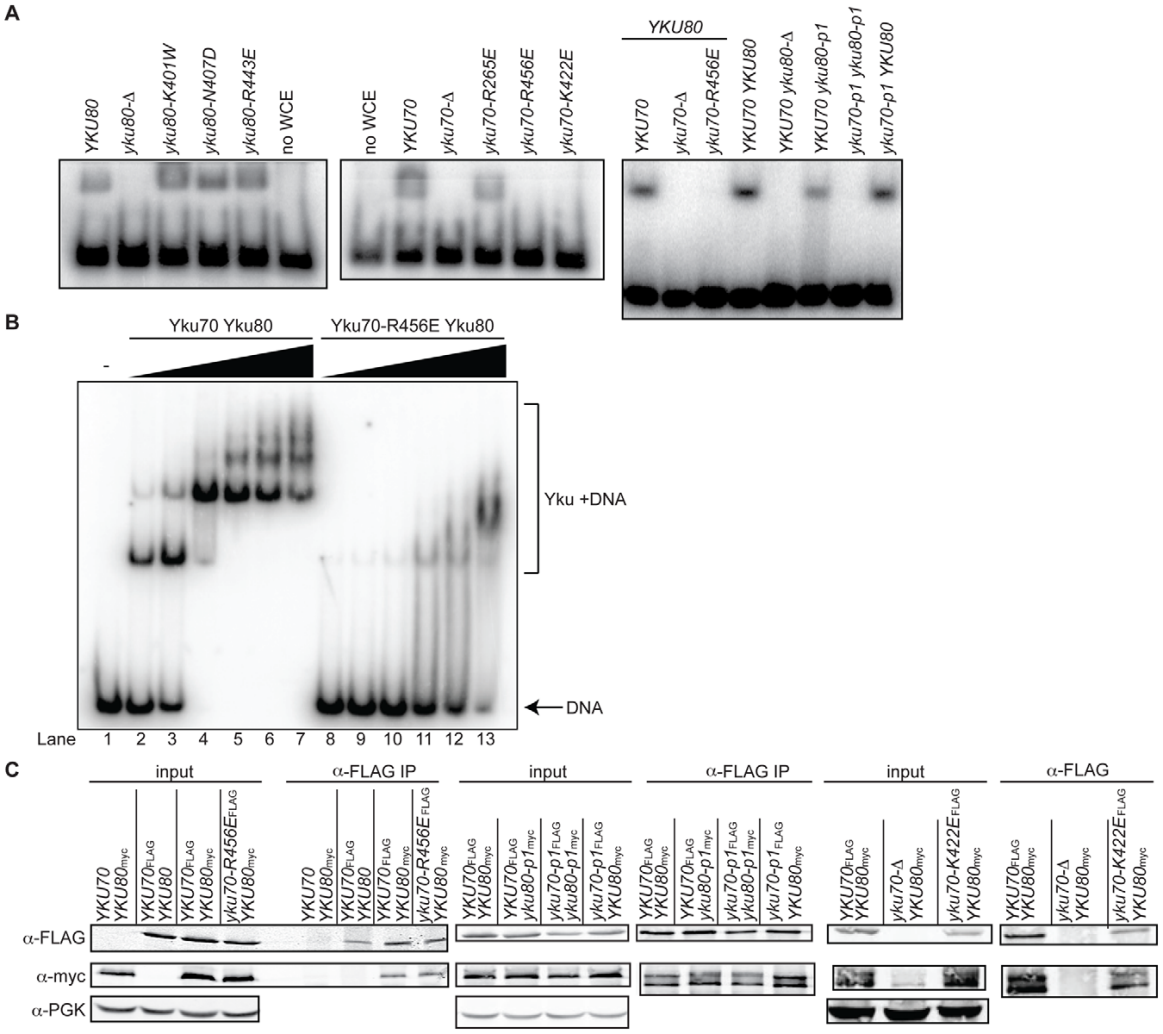
Generation of DNA end binding–defective Ku heterodimers. (A) DNA end binding assays using WCE. WCEs prepared from cells with the indicated genotypes were incubated with 1000-fold excess cold circular DNA and a ^32^P end-labeled nontelomeric 198 bp DNA fragment and run on a nondenaturing polyacrylamide gel. The left and middle panel strains were created by transformation of the respective WT or mutant *YKU70* or *YKU80* plasmid into *yku80-Δ* or *yku70-Δ* strains, whereas the right panel strains were created by plasmid co-transformation into *yku70-Δ yku80-Δ* double mutant strains. (B) DNA end binding assays using recombinant Ku heterodimers. His_6_-Yku70/His_6_-Yku80 and His_6_-Yku70-R456E/His_6_-Yku80 heterodimers were purified from Sf9 insect cells and the His_6_ tags cleaved. EMSAs were performed using a 198 bp ^32^P labeled nontelomeric DNA fragment at 0.67nM and 0 (lane 1), 0.15 (lanes 2 and 8), 0.5 (lanes 3 and 9), 1.5 (lanes 4 and 10), 5 (lanes 5 and 11), 15 (lanes 6 and 12), 50 (lanes 7 and 13) nM of WT or mutant Ku as indicated. (C) Assessment of steady state protein levels and heterodimerization of DNA end binding defective mutants. WT or mutant Yku70_FLAG_ was expressed in cells along with WT or mutant Yku80_myc18_. WCEs were prepared, α-FLAG IPs were performed and analyzed by western blotting as indicated. WCEs (input) and IP samples showed a doublet, which was Yku80_ myc18_-specific. α-PGK serves as a control for protein levels in the WCE prior to immunoprecipitation.

The impact of the mutations on DNA end binding was determined first by individually expressing each mutant allele in yeast lacking the corresponding wild type genomic copy and assaying DNA end binding activity in whole cell extracts (WCE) using an *in vitro* electrophoretic mobility shift assay (EMSA) [41]. In this assay, a 1000-fold molar excess of non-labeled closed circular DNA inhibits nonspecific binding to the 198 bp radiolabeled DNA fragment, resulting in an electrophoretic mobility shift of the radiolabeled fragment that is dependent on Ku binding to the DNA ends [41] (see also Figure 2A). As sought after, we saw significantly reduced end binding activity in two of the single missense mutant strains examined, *yku70-K422E* and *yku70-R456E*. Although Ku is known to bind DNA ends independently of other factors, we also examined purified recombinant WT and Yku70-R456E/Yku80 (Figure S1) heterodimers. We observed a 50- to 100-fold reduction in the DNA end binding affinity of the mutant (Figure 2B), confirming that the targeting indeed affected Ku's inherent ability to bind DNA ends.

As shown in Figure 1B, the *yku70-IMFQ266-269QDEY* and *yku80-SKKDS400-404DEEDD* mutations (referred to hereafter as *yku70-p1* and *yku80-p1*, respectively) targeted amino acids on opposing sides of the DNA binding channel. Whereas expression of *yku70-p1* with *YKU80* or *yku80-p1* with *YKU70* did not result in a severe deficiency in DNA end binding, expression of both panel mutant alleles resulted in undetectable DNA end binding activity in the WCE EMSA (Figure 2A and Figure S2). These results suggest that there are synergistic contributions to Ku's stable association with DNA ends mediated by amino acids on each side (i.e., leading and lagging) of the DNA binding channel.

Prior to utilizing the alleles identified as being DNA end binding defective for *in vivo* analyses, we needed to determine whether the loss of DNA end binding was the indirect result of markedly reduced steady state protein levels or loss of heterodimer formation. Using epitope tagged versions of Yku70 and Yku80, we found that the mutations did not substantially alter steady state protein levels (Figure 2C, input) or the ability of the mutant subunits to form heterodimers (Figure 2C, α-FLAG IP). Anti-FLAG immunoprecipitates from *yku70-R456E_FLAG_, yku70-p1_FLAG_*, and *yku80-p1_myc18_* single mutant WCE contained WT levels of both subunits, despite the presence of the mutation. The levels of Yku70-K422E_FLAG_ and Yku80_myc18_, and Yku70-p1_FLAG_ and Yku80-p1_myc18_ were only slightly reduced in the *yku70-K422E_FLAG_ YKU80_myc18_* single mutant, and *yku70-p1_FLAG_ yku80-p1_myc18_* double mutant strains' input and anti-FLAG IPs. Thus, the *yku70-K422E* and *yku70-R456E* single mutants and the combination of the *yku70-p1* and *yku80-p1* panel mutant alleles appeared to directly impact Ku's ability to bind DNA ends.

**Figure 3:**
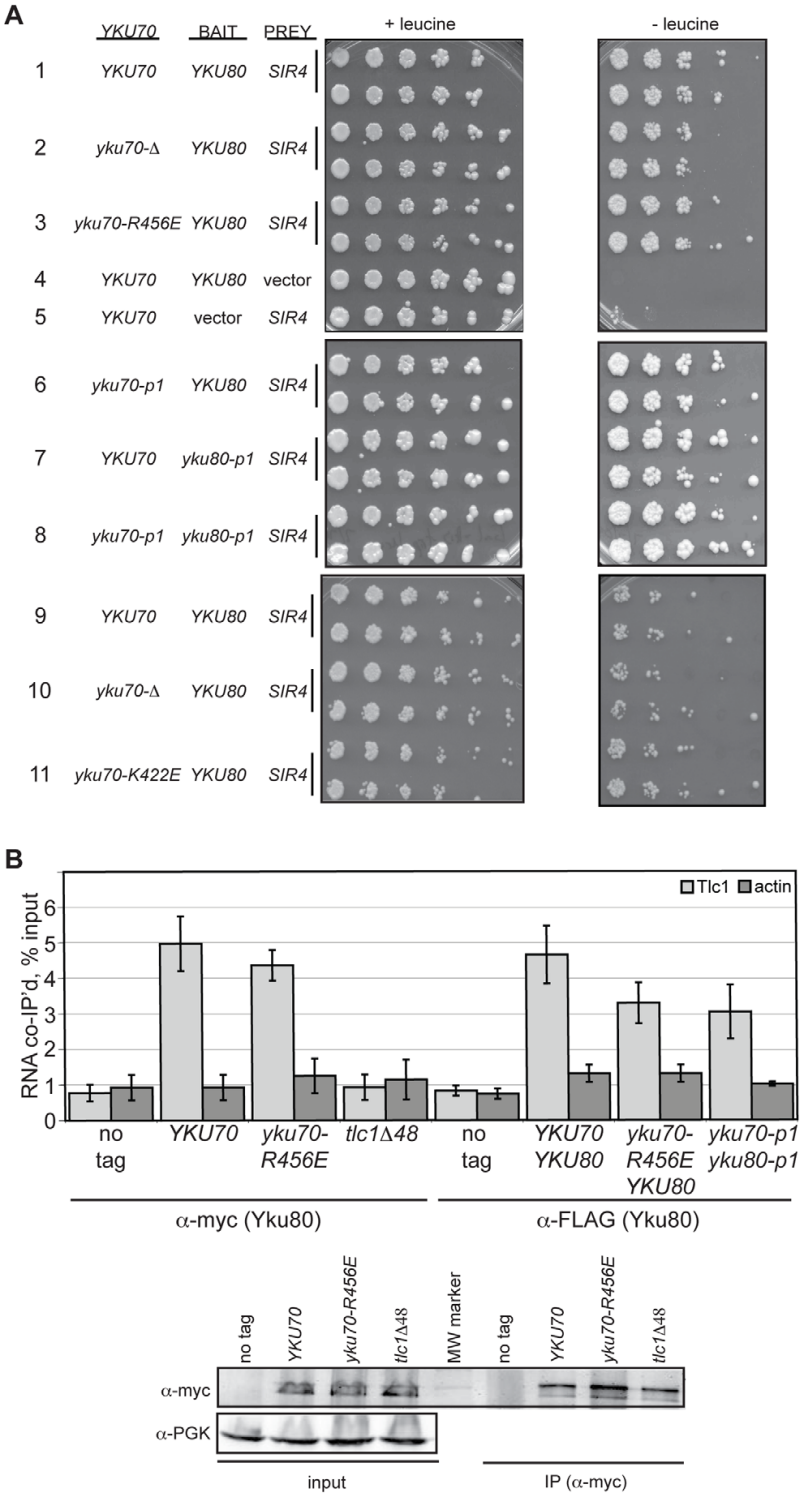
DNA end binding–defective Ku heterodimers interact with Sir4 and TLC1. (A) Yku80-Sir4 yeast-two-hybrid assay. YAB327 was transformed with indicated *YKU70* plasmid, a BAIT plasmid (*YKU80,* vector, or *yku80-p1*) and the PREY (*SIR4* or vector) plasmid. Serial dilutions of these strains were plated onto –His –Trp –Ura in the presence (+) or absence (–) of leucine. Interaction between Yku80 and Sir4 results in activation of a *LEU2* reporter and growth in the absence of leucine. (B) TLC1 co-immunoprecipitation. WCEs were prepared from strains with the indicated genotypes, α-myc or α-FLAG immunoprecipitations performed, RNA isolated, and TLC1 and actin RNA analyzed by reverse transcriptase RT-PCR. Reverse transcriptase RT-PCR was also carried out on WCEs prior to immunoprecipitation to control for RNA levels and calculate percent RNA co-immunoprecipitated. A bar graph shows the amount of TLC1 co-immunoprecipitated with myc- or FLAG-tagged Yku80. Error bars represent one standard deviation (SD). α-Myc western blot showing equal IP of myc-tagged Yku80 in *YKU70, yku70-R456E*, and *tlc1-Δ48* strains. α-PGK serves as a control for protein levels for the input WCEs prior to immunoprecipitation.

As with heterodimerization, the interpretation of our *in vivo* findings would be confounded if the mutations conferring a DNA end binding defect also disrupted Ku's ability to associate with the telomeric factor Sir4 or the telomerase subunit TLC1. Yeast Ku interacts with Sir4 via the Yku80 subunit [40]; therefore, we examined whether the Yku80-Sir4 interaction was retained in the DNA end binding defective mutants using a previously reported Yku80-Sir4 yeast two-hybrid system with a *LEU2* reporter [20]. Consistent with the prior observation that the Yku80-Sir4 yeast two-hybrid interaction was independent of Yku70 [20], we found that the presence of *yku70-R456E*, *yku70-p1* or *yku70-K422E* instead of *YKU70* did not impair the association between Yku80 and Sir4, as evidenced by growth of strains expressing these alleles, the *YKU80* BAIT, and the *SIR4* PREY on media lacking leucine (Figure 3A, compare strains 3 and 6 with 1, and 11 with 9). Significantly, the substitution of *yku80-p1* for *YKU80* on the BAIT plasmid in *YKU70* or *yku70-p1* cells did not inhibit growth in the absence of leucine (Figure 3A, compare strains 7 and 8 with 1). These results suggest that the mutations did not hinder the association between Yku80 and Sir4.

We also assayed the *in vivo* association of TLC1 with the Yku70-R456E Yku80 and Yku70-p1 Yku80-p1 Ku heterodimers. To do so, we immunoprecipitated mutant or WT Ku from asynchronous cultures expressing myc or FLAG tagged-Yku80 and performed real-time, reverse transcriptase polymerase chain reaction (RT-PCR) to detect TLC1 RNA. The *yku70-R456E* strain exhibited a level of TLC1 enrichment comparable to the *YKU70* strain in both myc- and FLAG-tagged *YKU80* backgrounds (Figure 3B; *P* = 0.51 and 0.20, respectively). Enrichment for *TLC1* was not seen in the ‘no tag’ control or in a *tlc1-Δ48* strain, which is defective for interaction with Yku80 [17](*P = *0.007). Likewise, actin RNA was not enriched in any of the reactions, demonstrating specificity of the co-immunoprecipitation. TLC1 was enriched similarly in *yku70-p1 yku80-p1_FLAG_* anti-FLAG IPs (*P* = 0.17). Thus, both of these DNA end binding defective Ku heterodimers were capable of binding TLC1 *in vivo*.

### DNA end binding–defective Ku heterodimers show decreased association with telomeric chromatin

**Figure 4:**
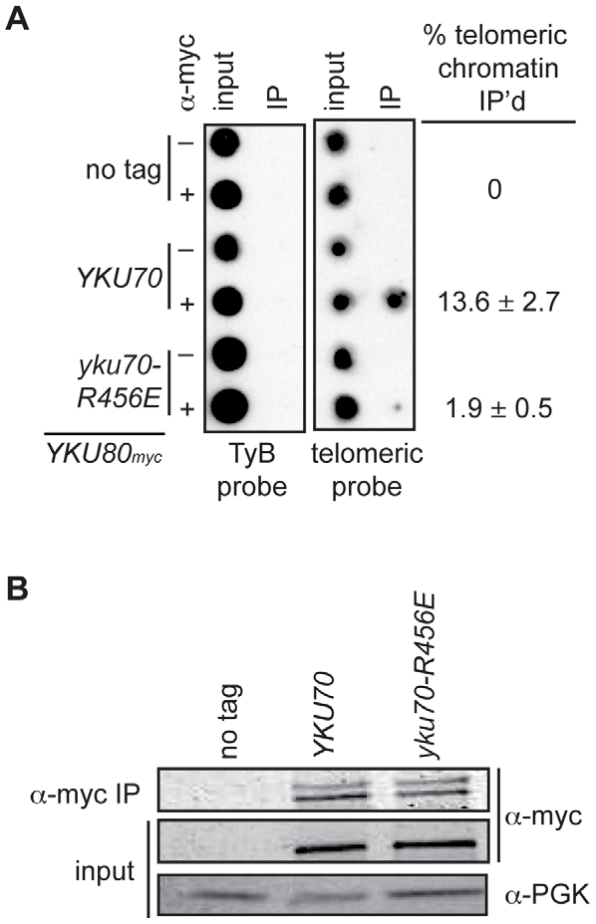
DNA end binding–defective Ku heterodimers show reduced association with telomeres. (A) Myc-tagged Yku80 was immunoprecipitated from sheared chromatin prepared from crosslinked cells containing Yku70 or Yku70-R456E. Isolated DNA was analyzed by dot-blot with a radiolabeled telomere specific probe or TyB probe for nonspecific association. The mean percentage of telomeric DNA immunoprecipitated in five trials is shown +/- one SD. (B) Western blot showing equivalent Yku80_myc18_ immunoprecipitation efficiency in the *YKU70* and *yku70-R456E* samples. α-PGK serves as a control for protein levels in input WCEs prior to immunoprecipitation.

The above analyses indicated that we had succeeded in introducing mutations into Ku that markedly impacted on DNA end binding without substantially altering protein stability, heterodimerization or interactions with known telomeric factors [40]. Therefore, we proceeded to address whether selective loss of Ku's DNA end binding activity altered its association with telomeric chromatin *in vivo* by performing chromatin immunoprecipitation (ChIP) assays. We found an 80–86% decrease in the amount of telomeric DNA that immunoprecipitated with Yku80_myc_ in the *yku70-R456E* strain relative to the amount immunoprecipitated in the *YKU70* strain (Figure 4A and Figure S3A). This decrease was not due to decreased levels of Yku80_myc_ or decreased immunoprecipitation efficiency, as protein levels appeared equal in both the input and α-myc IP samples (Figure 4B and Figure S3B). We also performed ChIP experiments with the *yku70-p1 yku80-p1* double panel mutant strain (Figure S3A), which showed undetectable DNA end binding in the WCE EMSA (Figure 2A). The immunoprecipitates isolated from the double panel mutant contained less than 1% of the telomeric DNA isolated from WT immunoprecipitates. However, we could not rule out the possibility that this was due to the reduced immunoprecipitation efficiency that was observed in this mutant under these conditions (Figure S3B). Thus, we conclude that, at the very least, the vast majority of Ku's localization to telomeres requires that it bind directly to telomeric ends.

### Loss of Ku's DNA end binding activity results in the loss of telomere end protection

**Figure 5:**
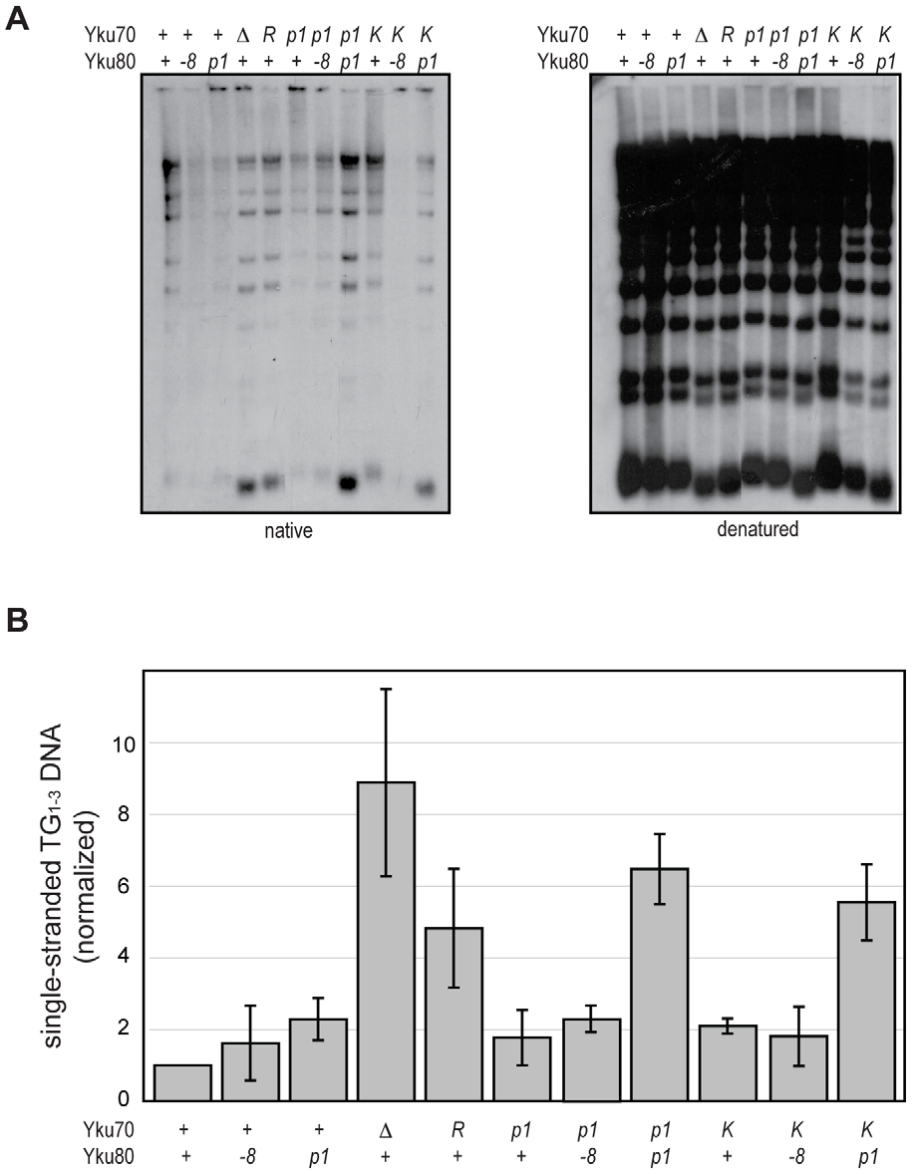
DNA end binding–defective mutants are deficient for telomeric end protection. (A) Telomeric G-overhang assay. XhoI digested genomic DNA fragments isolated from *yku70-Δ yku80-Δ* strains transformed with *CEN* plasmids containing the indicated alleles (+, WT; *Δ*, empty vector; R, *yku70-R456E; p1, yku70-p1* for top row or *yku80-p1* for bottom row; K, *yku70-K422E*; and -8, *yku80-*8) of *YKU70* and *YKU80* were incubated with a radiolabeled telomere-specific oligomeric probe and run on a native gel (left). The amount of single-stranded telomeric DNA was quantitated before the gel was denatured and probed with the same telomere probe to reveal the total amount of telomeric DNA (right). (B) Ratio of single-stranded telomeric DNA to total telomeric DNA, normalized to WT. Values represent the average of three independent experiments. Error bars indicate one SD.

An important role of Ku at telomeres is to protect the C-rich telomeric strand from exonucleolytic degradation by Exo1. The mechanism by which this is accomplished has not been directly studied; however, the simplest model posits that Ku sterically hinders Exo1's access and/or progression by loading directly onto telomeric ends. By examining the telomeric G-overhangs in the DNA end binding defective strains, we could now test this model directly. To do this, XhoI-digested genomic DNA prepared from asynchronous cultures was hybridized with a C-rich radiolabeled telomeric probe and subjected to native gel electrophoresis as previously described [34] (Figure 5A, native). The signal was quantified and normalized to the total telomeric G-strand DNA signal, which was obtained by in-gel DNA denaturation followed by hybridization with the same probe (Figure 5A, denatured, and 5B). Two of the DNA end binding defective Ku mutants, *yku70-R456E* and *yku70-p1 yku80-p1*, exhibited readily detectable ss G-rich DNA signals similar to a *yku70-Δ* strain. Thus, a loss of DNA end binding correlated with a loss of telomere end protection.

In apparent contradiction to this correlation, the *yku70-K422E* mutant strain, which was also defective in DNA end binding in the WCE EMSA, exhibited only a slight increase in ss G-rich DNA, comparable to strains expressing *yku70-p1* or *yku80-p1* (Figure 5). This could indicate that Ku's end binding activity is, in fact, not required for Ku's telomere end protection function. However, *yku70-K422E* single mutants also appeared similar to WT in all additional assays performed (telomere length, telomeric silencing, synthetic lethality with *tlc1-Δ*, temperature sensitivity, and imprecise NHEJ; Figure S4 and data not shown). It was possible that this mutant protein was unfolded *in vitro* or differentially sensitive to the WCE EMSA conditions. An alternative explanation was that the Yku70-K422E mutant protein, with its mutated residue on the lagging face of the DNA binding channel (Figure 1), was stabilized at telomeric ends *in vivo*. We reasoned that if this was the case then the combination of the *yku70-K422E* allele with the *yku80-p1* allele, whose mutated residues are on the loading face of the DNA binding channel (Figure 1), might destabilize the mutant Yku70-K422E-containing Ku's association with DNA ends *in vivo* and give rise to defective telomere end protection. Indeed, we found a synergistic increase in the ss G-rich DNA signal in the *yku70-K422E yku80-p1* double mutant strain (Figure 5). An increase was not observed, however, when *yku70-K422E* was combined with the *yku80-8* allele, which contains a mutated residue on Ku's loading face. This mutation impairs Ku's ability to bind Sir4 [40], but it does not affect Ku's ability to bind DNA ends (Figure S5). These findings suggest that the ability of Ku to protect telomeres from exonucleolytic resection requires that Ku bind directly to the telomeric end.

### Loss of Ku's DNA end binding activity results in short telomeres

**Figure 6:**
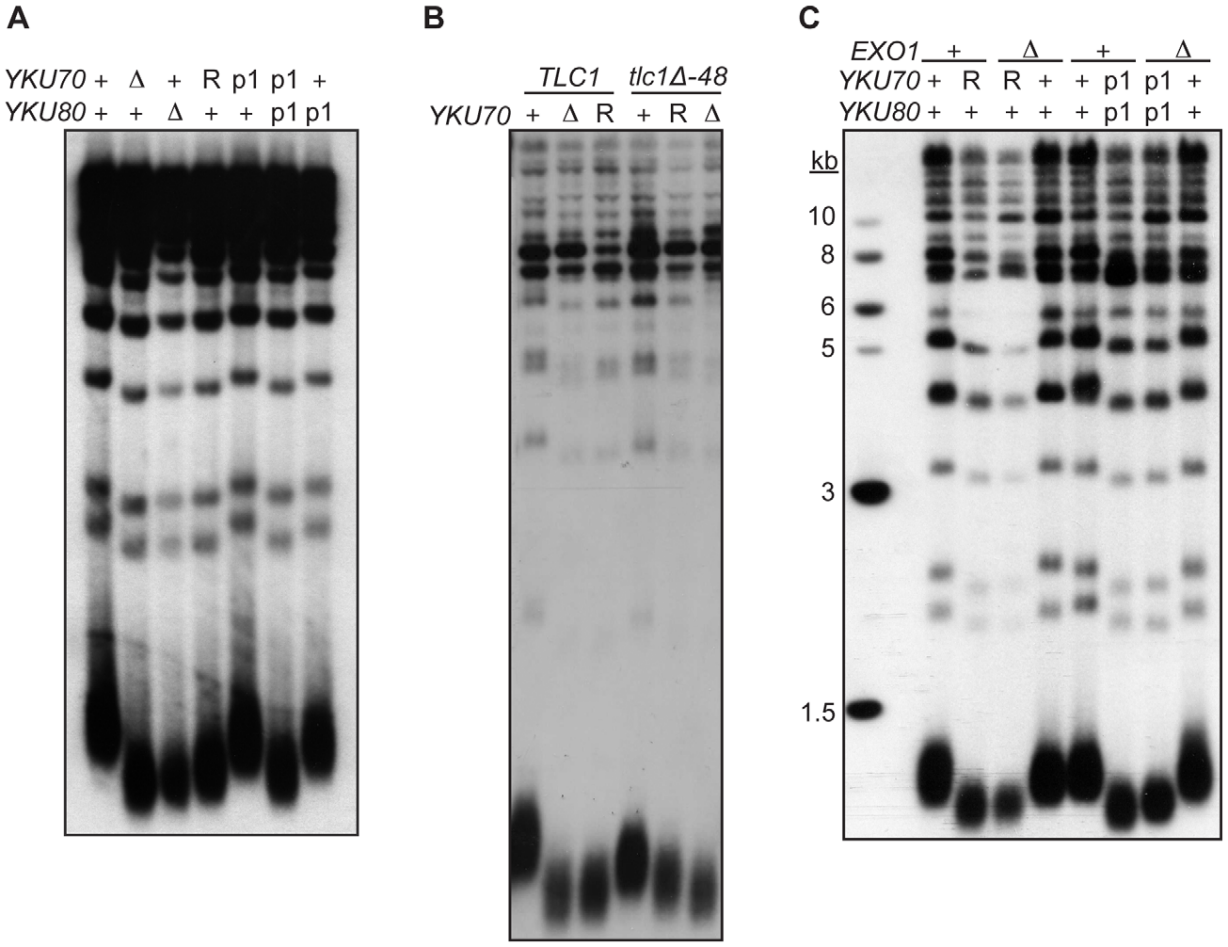
DNA end binding–defective mutants are deficient for telomeric length regulation. (A) XhoI digested genomic DNA, isolated from *yku70-Δ yku80-Δ* strain co-transformed with the indicated *CEN* plasmids (+,*YKU70* for top row or *YKU80*, for bottom row; *Δ*, empty vector; R, *yku70-R456E; p1, yku70-p1* for top row or *yku80-p1* for bottom row), was blotted and probed with an end labeled telomere specific oligo. (B) As in A, except the DNA was isolated from a *yku70-Δ tlc1-Δ* strain containing *YKU70*, *yku70-R456E*, or vector, and *TLC1* or *tlc1-Δ48 CEN* plasmids to generate the genotypes as indicated. (C) As in A, except indicated strains are either WT (+) or null (*Δ*) for the genomic copy of *EXO1*.

As described earlier, a second important telomeric function of Ku is in the maintenance of telomere length. Whereas it is known that this requires Ku's interaction with TLC1, it is unknown whether it requires Ku's direct association with telomeric ends. To address this question, we determined the length of telomeres present in *yku70-R456E* and *yku70-p1 yku80-p1* mutant strains. We found that the telomeres in these mutants were nearly as short as those in *yku70-Δ* and *yku80-Δ* null strains (Figure 6A). In contrast, the telomeres in the *yku70-p1* and *yku80-p1* single mutants were of WT length or only slightly shortened. Additionally, as illustrated in the *yku70-R456E* mutant, the degree of shortening was much greater than in a *tlc1-Δ48* mutant, in which Ku can no longer bind TLC1. Moreover, the telomeres were no shorter in a *yku70-R456E tlc1-Δ48* double mutant than in a *yku70-R456E* single mutant (Figure 6B). Therefore, and consistent with the TLC1 co-immunoprecipitation results (Figure 3B), the shortening observed in these DNA end binding defective Ku mutants was due to a defect downstream of TLC1 association.

These data suggest that Ku must load onto and stably associate with the telomere end in order to mediate its role in telomere length regulation, yet it was possible that the telomere length defect in the DNA end binding defective strains was due to the increased single-strandedness of the G-rich telomeric strand. If the failure to protect telomeric ends was the sole contributing factor to the shortened telomeres present in the end binding defective Ku mutants, then the deletion of *EXO1* should fully restore the length of the telomeres. We found that, although the deletion of *EXO1* fully complemented the defect in end protection of DNA end binding defective mutants (Figure S6), it did not fully restore telomeres to a WT length (Figure 6C). Thus, Ku's ability to bind telomeric ends contributes directly to the maintenance of telomere length beyond simple telomeric end protection.

### Loss of Ku's DNA end binding activity results in the loss of telomeric silencing

**Figure 7:**
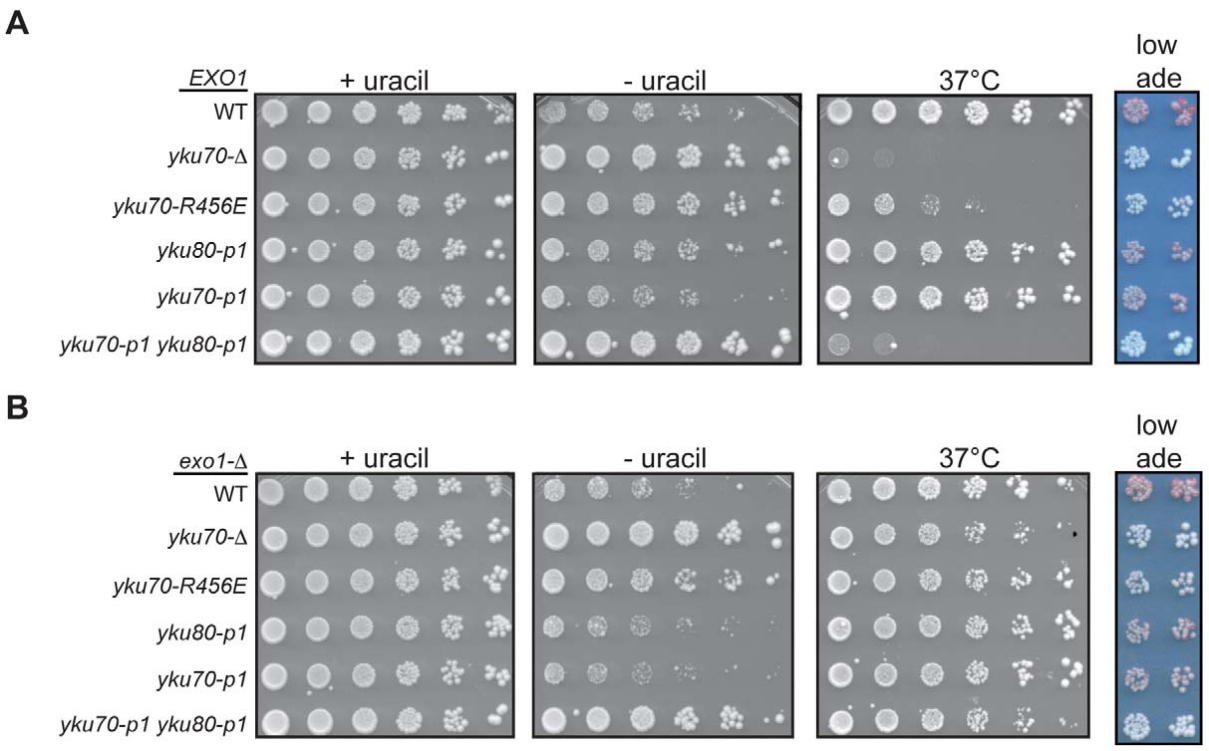
DNA end binding–defective mutants are deficient for telomeric silencing. (A) Telomeric silencing assay in DNA end binding defective strains. Shown are five-fold serial dilutions of an *EXO1 yku70-Δ yku80-Δ* strain with VII-L *URA3* and V-R *ADE2* telomeric reporters (YAB219) transformed with plasmids containing vector, WT, or mutant versions of *YKU70* (*TRP1*) and *YKU80* (*LEU2*). Growth was monitored on –Trp –Leu –Ura at 28°C to examine de-repression of *URA3* (-uracil plates) and on –Trp –Leu at 28°C to monitor plating efficiency (+uracil plates) and 37°C as a surrogate marker for telomere end protection (37°C plates) [26]. Growth was also examined on –Trp –Leu media with limiting Ade to examine de-repression of *ADE2* (low ade plates). (B) Telomeric silencing assay in DNA end binding defective strains lacking *EXO1.* Experiment was performed as described in A, except the plasmids were transformed into an *exo1-Δ yku70-Δ yku80-Δ* telomeric reporter strain (YAB353).

Whereas we predicted Ku's association with the telomeric end would be required for its role in telomere end protection and length regulation, a role for Ku's DNA end binding in the formation of subtelomeric heterochromatin was not as clearly anticipated [30], [42], [43]. In the formation of subtelomeric chromatin, Ku is recruited to the centromere-proximal region of subtelomeric DNA in a Sir4-dependent manner [25]. Therefore, the DNA end binding activity of Ku may not be required for Ku's ability to promote the silencing of subtelomeric reporters. To address this question, we utilized a strain containing a *URA3* telomeric reporter gene, which is subject to Ku-dependent silencing [43], to examine whether the DNA end binding mutations resulted in telomeric silencing defects. WT strains grow poorly on medium lacking uracil (and robustly on 5-FOA medium) because of the spread of telomeric heterochromatin and secondary transcriptional repression of the adjacent *URA3* gene. Cells with a silencing deficiency, such as *yku70-Δ*, display the opposite growth pattern – robust growth in the absence of uracil and minimal growth in the presence of 5-FOA. As in the previous assays of Ku's telomere end protection and length maintenance functions, the *yku70-p1 yku80-p1* double mutant strain exhibited a markedly defective phenotype, whereas the single panel mutant strains, *yku70-p1* and *yku80-p1*, displayed a phenotype similar to that of WT (Figure 7A). The silencing defect in the *yku70-R456E* mutant was not as severe as the defect observed in the *yku70-Δ* or double panel mutant strain, but was clearly evident by the larger colony size and greater colony number on the plate lacking uracil (Figure 7A). This partial silencing defect was also evident when we examined the expression of a second telomeric reporter gene in this strain, *ADE2*. The reversible nature of telomeric silencing results in variegation, such that WT colonies are mostly red (due to *ADE2* repression) with some white sectors (due to *ADE2* expression). However, colonies from strains that are deficient in telomeric silencing are white in appearance. Utilizing this reporter, we found that the double-panel mutant and *yku70-Δ* strains were white, hence, defective for telomeric silencing (Figure 7A). The *yku70-R456E* strain exhibited a very slight variegated appearance, suggesting that it was not completely defective in silencing; however, it was clearly dysfunctional when compared to WT, *yku70-p1*, and *yku80-p1* strains.

Rap1 plays a central role in the establishment of telomeric silencing by binding ds telomeric repeats and recruiting the essential silencing proteins Sir3 and Sir4 to chromatin [8]. Because excess degradation of the telomeric sequence would result in the loss of Rap1 binding sites, the telomeric silencing defect of the DNA end binding defective Ku mutant strains could be an indirect consequence of their defect in telomere end protection. To determine if such was the case, we repeated the silencing assay in an *exo1-Δ* background. Deletion of *EXO1* restored the level of ss, G-rich DNA in the DNA end binding defective mutants to near WT levels (Figure S6), which was further reflected by suppression of the temperature sensitive phenotype (Figure 7B). Despite restoring ds telomeric DNA, we found that deleting *EXO1* only slightly restored silencing, as evidenced by the slightly decreased growth of *yku70-R456E exo1-Δ* and *yku70-p1 yku80-p1 exo1-Δ* strains on medium lacking uracil relative to their corresponding *EXO1* strains (compare Figure 7B to 7A). Similarly, the deletion of *EXO1* led to the appearance of very faint pink colonies in the DNA end binding defective mutants (Figure 7B). Altogether, these results indicate that the DNA end binding activity of Ku (and, hence, its direct association with telomeric DNA) is required for its role in telomeric silencing.

### Loss of Ku's DNA end binding activity results in increased imprecise NHEJ

In each of the telomeric function assays performed, the *yku70-R456E* and *yku70-p1 yku80-p1* mutants exhibited loss of function similar to that of *yku70-Δ* or *yku80-Δ* strains; however, the interaction assays (e.g., for heterodimerization, TLC1 interaction, and Yku80-Sir4 yeast two-hybrid interaction) argued against a global null phenotype. This was further revealed in an assay for imprecise NHEJ in which the DNA end binding defective mutants could be distinguished clearly from Ku-deficient strains. The yeast strain used in this assay contains an HO endonuclease cleavage site at the mating type (*MAT*) locus but lacks *HML* and *HMR* donor sequences, which are required for the normal homology directed repair of HO-cleaved *MAT* [44]. The expression of HO endonuclease, and, hence, the induction of a DSB, is under the control of a galactose-inducible promoter. Because of the absence of a homologous donor sequence, only NHEJ can repair a DSB created by HO at *MAT* in this strain. When cells are plated on galactose-containing medium, HO is constitutively expressed and available to re-cleave any accurately repaired cleavage sites, precluding cell growth. However, when the HO cleavage site is altered by an imprecise end-joining event, a colony can form. This imprecise NHEJ occurs in approximately 0.1% of plated WT cells [44].

**Figure 8:**
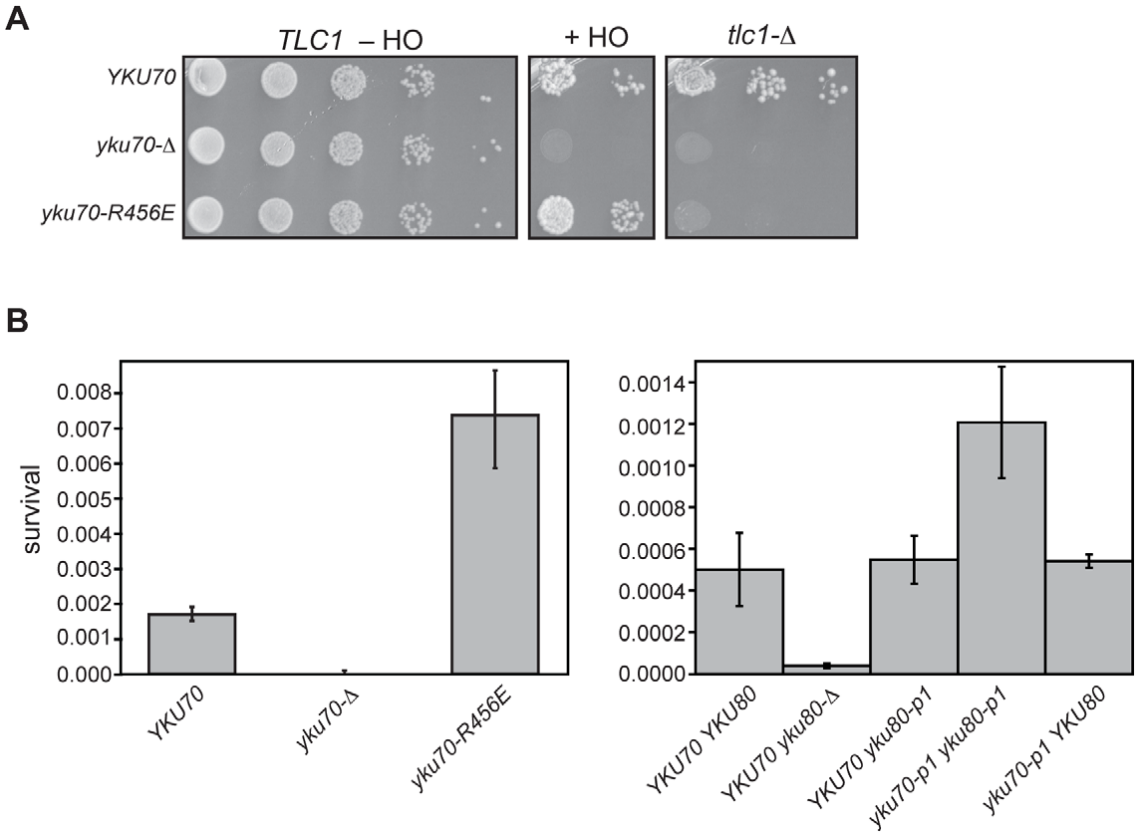
DNA end binding–defective mutant Ku strains can be distinguished from Ku-deficient and Ku WT strains in an assay for error prone end-joining. *(A) Imprecise NHEJ and synthetic lethality assay using strains with HO endonuclease under the control of a galactose-inducible promoter and a single HO cleavage site repairable by NHEJ only. Serial dilutions of YAB199 (yku70-Δ tlc1-Δ/pTLC1 URA3*) containing a *YKU70*, vector, or *yku70-R456E CEN* plasmid were spotted onto –Trp –Ura plates to monitor plating efficiency (TLC1 –HO), -Trp –Ura plus galactose plates to assay error prone end-joining (+HO) or –Trp plus 5-FOA plates to assay synthetic lethality in the absence of telomerase (*tlc1-Δ*). (B) Quantitative imprecise NHEJ assays. Left: YAB198 (*yku70-Δ*) containing *YKU70*, *yku70-p1, yku70-R456E,* or vector (*TRP1*) were plated on –Trp media containing galactose and –Trp media to determine colony survival under galactose-induced constitutive HO expression. Averages of 4 independent experiments are shown with error bars showing one SD. Right: Same as Left except the strains analyzed were YAB273 (*yku70-Δ yku80-Δ*) containing *YKU70*, *yku70-p1,* or vector (*TRP1*) and *YKU80* or *yku80-p1* (*LEU2*) plasmids.

Previous studies have demonstrated that colony survival in the presence of constitutively expressed HO (i.e., imprecise NHEJ) is reduced to 0.0001% of cells plated in the absence of Ku [44]; therefore, we expected to observe little colony formation when the DNA end binding defective Ku mutations were examined in this assay. However, unlike the *yku70-Δ* strain, the *yku70-R456E* strain gave rise to ample colonies when grown on galactose (Figure 8A, +HO). This strain also was engineered to test whether the *yku70-R456E* mutation impacted cell viability in the absence of *TLC1* (and, therefore, telomerase), which requires Ku's telomere end protection function [40]. In contrast to the result of the imprecise NHEJ assay, the effect of *yku70-R456E* mutation on survival in the absence of telomerase was similar to that of a *yku70-Δ* mutation because both resulted in synthetic lethality when combined with a *tlc1-Δ* mutation (Figure 8A, *tlc1-Δ*). Thus, the seemingly WT behavior of the *yku70-R456E* mutant in the HO assay (versus the more null-like behavior in the telomere function assays mentioned above) was not due to a difference in the general requirement for Ku in the different strain backgrounds.

We noted that the *yku70-R456E* mutants appeared to grow more robustly than WT when plated as serial dilutions on galactose-containing medium (Figure 8A, +HO). To confirm this, as well as to test the *yku70-p1 yku80-p1* double panel mutant for this phenotype, we repeated the assay in a strain containing an intact chromosomal copy of *TLC1*. We consistently found that both DNA end binding defective mutants displayed greater than WT number of colonies on galactose-containing medium (Figure 8B). The *yku70-Δ* mutant, in contrast, showed minimal growth on galactose. Additionally, as with the telomeric assays, *yku70-p1* and *yku80-p1* single mutant strains behaved like WT with comparable levels of survival (Figure 8B). Therefore, the phenotype of the DNA end binding defective mutants was different not only from the *yku70-Δ* strain, but also from WT. Taken together, these results provide phenotypic evidence that argue against the telomeric phenotypes simply being a reflection of a generalized Ku deficiency.

## Discussion

We found that the singular loss of Ku's ability to bind DNA ends had dramatic effects on its association with telomeric chromatin and its telomeric functions. These results demonstrate that Ku predominantly associates with telomeres via direct DNA end binding and that DNA end binding is required for Ku to carry out its various telomeric functions. Thus, Ku is an integral component of the telomere cap.

So, what is involved in this integral association between Ku and telomeric DNA? The phenotypes seen with the DNA end binding defective mutants and the position of these mutations within the Ku heterodimer provide clues that address this question. There appear to be at least two distinct steps involved: the initial loading of Ku onto the telomeric end, and the stabilization of Ku at telomeres. For example, the *yku70-R456E* mutant contains a single amino acid substitution on the loading side of the central channel (Figure 1). More telomeric chromatin is associated with Yku70-R456E Yku80 heterodimers (Figure 4) than one would expect based upon *in vitro* DNA end binding assays with recombinant protein (Figure 2B). This is likely due to the differences in substrates in the two assays – naked DNA fragments versus telomeres, and these data suggest that, once this mutant Ku heterodimer is able to load onto telomeric ends *in vivo*, a fraction can be stabilized. *In vitro*, this mutant protein is not as stably bound to DNA as WT, as suggested by the excessive smearing seen in the EMSA (Figure 2B). The synergistic loss of telomeric function and synergistic increase in imprecise NHEJ observed with the *yku70-K422E yku80-p1* double versus the *yku70-K422E* and *yku80-p1* single mutants (Figure 5 and Figure S4) also points to factors influencing the stabilization of Ku's binding to DNA ends *in vivo* relative to the *in vitro* assays.

The loss of Ku's ability to bind DNA ends affected each of the telomeric functions examined in this study. Least surprisingly, loss of DNA end binding resulted in a telomere end protection defect, with an *EXO1*-dependent increase in G-overhang DNA as observed in *yku-Δ* strains (Figure 5 and Figure S6). These data are consistent with Ku's DNA end binding contributing to end protection through direct steric hindrance of Exo1. However, the mechanism of Ku-mediated telomere end protection is likely to be more complex. Despite the association of Yku80 (and presumably Yku70) with telomeres throughout the cell cycle [17], its role in the protection of *de novo* telomeres is limited to G1 [45], a time when the Cdc13-Stn1-Ten1 (CST) complex is dispensable [46]. This may be the case at native telomeres as well [45]. Thus, Ku or telomeres may be altered as cells progress through S phase, after which telomeres no longer require Ku, but rather depend on the CST complex, as well as Rap1, Rif1 and Rif2, for their end protection [45], [46]. That end binding is required for Ku's role in telomere end protection is also consistent with the observation that Ku is required to prevent the initiation of telomere resection [45].

Ku has been shown to repress recombination near telomeres. It has been proposed that this repression is mediated through Ku's promotion of a telomere fold-back architecture via interactions with the subtelomeric core X element [47], [48]. Such an architecture may also serve to protect telomeres from end resection. Such a structure is likely to unfold with progression through S phase and telomere replication, perhaps limiting its role to G1. This opening of the fold-back structure is consistent with the greater amount of sub-telomeric DNA found in Yku80 ChIPs from cells in G1 compared to the amount seen after cells have entered S phase [17]. Determining whether the DNA end binding Ku mutants retain their interaction with core X and/or repress recombination near telomeres will be important toward our understanding of the significance of Ku – core X interactions.

In contrast to a defect in telomere end protection, the defect in telomeric silencing observed in the DNA end binding defective mutants (Figure 7A) was not necessarily expected for two reasons. First, Ku does not impact on Sir4′s association with telomeric repeat-associated chromatin, but rather on its association with subtelomeric nucleosomal chromatin [33]. Second, the association of Ku with subtelomeric chromatin is diminished in the absence of Sir4, suggesting that Ku is recruited to the subtelomere via its association with Sir4 [25]. How, then, might Ku's association with the very end affect subtelomeric chromatin? The answer to this question may also relate to a role for Ku in mediating a fold-back structure at telomeres and the importance of this structure for the formation or maintenance of silent telomeric chromatin [47]–[50]. We note that, although, the truncated Tel V-R and Tel VII-L telomeres used in our silencing assays lack core X elements, Ku has been shown to associate with non-core X sequences near telomeres and has core X-independent effects on recombination repression [47], [48].

Ku also contributes to the tethering of some telomeres to the nuclear periphery, a phenomenon that contributes to telomeric silencing [43], [51]. Thus, it is possible (and indeed likely) that the Ku DNA end binding mutations, which alter Ku's association with telomeres, also impact on the localization of some telomeres to the nuclear envelope. Therefore, might the effects these mutations have on telomeric silencing be a consequence of loss of telomere perinuclear localization? Although we can not eliminate this possibility at those native telomeres that are subjected to both Ku-mediated localization and silencing, the perinuclear anchoring of the truncated Tel V-R and Tel VII-L telomeres used in our telomeric silencing assays is not Ku-dependent [38], [52]. Therefore, the results from this assay reveal that the silencing defects observed in DNA end binding defective mutants are independent of Ku's role in nuclear tethering of telomeres.

The short telomere phenotype seen in Ku DNA end binding defective mutants suggests that the association of Ku with telomeric ends is also essential for Ku's ability to positively regulate telomere length. Ku-TLC1 association was not significantly diminished in the DNA end binding defective mutants (Figure 3B), arguing against this simple explanation for the short telomeres in these mutants (Figure 6A). Moreover, *yku70-R456E* mutants had telomeres that were significantly shorter than the telomeres present in *tlc1-Δ48* mutants (Figure 6B). These results indicate that binding TLC1 (and presumably telomerase) alone is insufficient and that end binding is essential for Ku-mediated telomere length regulation. Deletion of *EXO1*, while suppressing the increased ss G-strand telomeric DNA, had only minor effects on the short telomere phenotype, arguing against the loss of end protection in the end binding defective mutants as the primary contributor to the loss of telomere length (Figure 6C). Therefore, we conclude that the direct association of Ku with the very end is required for Ku to contribute to telomere length maintenance.

By targeting residues in Ku that were predicted to contact DNA, we generated Ku heterodimers that were defective for DNA end binding yet proficient for TLC1 association *in vivo*. These mutants are the reverse complement of the *yku80-135i* mutant, which is defective for its ability to bind TLC1 but fully competent for binding DNA ends [24]. These data suggest that Ku's association with TLC1 does not involve the central channel and that Ku has separate determinants for binding DNA ends and TLC1. Whether Ku can simultaneously bind telomeric DNA and TLC1 RNA has yet to be determined and would have implications for molecular models of how Ku influences telomere length regulation.

We observed that strains that were defective in DNA end binding did not phenocopy Ku-deficient strains in an assay for imprecise end-joining (Figure 8). This result supports the conclusion that the telomere defects in these strains were not due to a generalized deficiency in function. Quantification of survival under constant DSB-inducing conditions, and subsequent sequencing of the repaired junctions, revealed that the DNA end binding defective mutants exhibited an increase in survival relative to WT and that the repaired junctions were distinct from junctions observed in WT and null strains (Figure 8 and data not shown). Thus, these mutants exhibit a novel phenotype. This likely relates to the fact that, in contrast to *yku70-Δ* or *yku80-Δ* strains, Ku is still present in these mutants; in contrast to WT strains, in which Ku is also present, the Ku that is present exhibits markedly reduced binding to DSBs. How the presence of these mutant proteins might result in more efficient re-ligation through an error-prone end-joining pathway is unknown. One possibility is that they have retained not only their interactions with telomeric factors (such as Sir4 and TLC1 as we have shown), but also with NHEJ factors. Consequently, DNA end binding defective Ku heterodimers might titrate one or more factors away from DSBs, which results in an increase in Ku-independent error-prone NHEJ.

Our findings show that Ku must access and load onto telomeric ends in order for it to participate in its various telomeric functions in *S. cerevisiae*. It will be interesting to determine whether Ku's end binding property is also required at human telomeres. Ku in human cells has been shown to prevent the rapid loss of telomeric repeats and the generation of so-called t-circles (extrachromosomal circular DNA containing telomeric repeats) [53]. Perhaps in human cells, Ku plays a role in stabilizing the t-loop structure, similar to its proposed role in maintaining the protective fold-back architecture of yeast telomeres.

## Materials and Methods

### Strains and plasmids

The strains and plasmids used in this study are described in Table S1 and Table S2, respectively. Strains lacking *YKU70* and/or *YKU80* were constructed using one-step allele replacement with the indicated selection marker. YAB285, YAB438 and YAB439 were constructed by integrating *yku70-R456E*, *yku70-p1* and *yku80-p1* into YTSF79 [17] by pop-in/pop-out technique. Mutations were introduced into *YKU70* and *YKU80* containing plasmids using oligonucleotide single-stranded mutagenesis as described [40].

### Electrophoretic mobility shift assays (EMSA)

For EMSA using yeast WCEs, cell lysates were prepared, and EMSA was performed using a modification of a previously published protocol [41]. Five ml cultures were lysed with glass beads in lysis buffer [420 mM KCl, 42 mM HEPES, 4.2 mM EDTA, 0.1 mM dithiothreitol (DTT)]. Approximately 10–15 µg of protein from each cell lysate was incubated with (1.5 ng) ^32^P end-labeled Bgl II – Nru I fragment (198 bp) of pcDNA3.1 (Invitrogen) for 15 minutes at room temperature, along with 1 µg unlabeled closed circular pcDNA3.1. The samples were loaded onto a 5% polyacrylamide gel in Tris-glycine buffer and run at 30 mA for approximately 45 minutes. The gel was dried and exposed to film or a phosphorimager screen and analyzed using a Storm865 imaging system (Molecular Dynamics). Quantitation of the phosphorimager image was done using ImageQuant software.

For EMSA using recombinant Ku, binding reactions were prepared containing 20 mM Tris-HCl pH 8, 50 mM NaCl, 1 mM DTT, 5 mM MgCl_2_, 0.5 mM EDTA, 100 µg/ml BSA, 11% glycerol, 1.5 ng of ^32^P- labeled DNA (as used for the yeast WCE EMSA), and the indicated amounts of purified recombinant Ku. Reactions were incubated for 15 minutes at room temperature. The reactions were loaded onto a 5% polyacrylamide gel, which was prerun at 40 mA for 30 minutes. Electrophoresis was carried out in Tris-glycine buffer at 40 mA for 100 minutes. The gel was dried and analyzed as above.

### Expression and purification of recombinant yeast Ku

Ku70, Ku70-R456E and Ku80 were individually subcloned into pFastBac HT A (Invitrogen) opened with NcoI and NotI. An N-terminal 6xHis tag followed by a linker region and a TEV protease recognition sequence precedes the multiple cloning site.

Individual recombinant baculoviruses expressing either Ku70, Ku70-R456E or Ku80 were made from the bacmids and used to infect Sf9 cells by the Baylor Baculovirus Core. Sf9 cell stocks (Invitrogen) were maintained as suspension cultures in spinner vessels prior to infection. Sf9 cells were co-infected at a MOI of 1 for each recombinant virus for 48 hours in a 1 liter spinner vessel in 500 ml of Grace's Insect Medium (Invitrogen) + 10% FBS at a density of 1.0×10^6^ cells/ml. At 48 hours post-infection, the cells were centrifuged at 800× g for 10 minutes, washed one time in PBS, and the cell pellets were immediately placed in a −80°C freezer for storage.

Cells were disrupted by the addition of lysis buffer (50 mM phosphate buffer pH 8.0, 300 mM NaCl, 0.05% Tween 20, 5 mM β-mercaptoethanol, 10 mM imidazole). The lysates were incubated on ice for 30–60 minutes and then sonicated on ice using a Misonix sonicator probe. The lysate was cleared by ultracentrifugation at 26K rpm, 4°C for 15 minutes and then incubated with Ni-NTA agarose (Qiagen) rotating for 2 hours at 4°C. The resin was then washed twice in buffer (50 mM phosphate buffer pH 8.0, 300 mM NaCl, 5 mM β-mercaptoethanol, 20 mM imidazole). Recombinant Ku was eluted in buffer (50 mM phosphate buffer pH 8.0, 300 mM NaCl, 5 mM β-mercaptoethanol, 250 mM imidazole). The Ku containing eluates were identified by SDS-polyacrylamide gel electrophoresis (PAGE) and Coomassie stain, then combined and concentrated on an 50NMDL Amicon filter (Millipore). The Ku retentate was then put over a Superdex 200 16/60 sizing column and Ku dimer containing eluates were concentrated on a 50NMDL Amicon filter (Millipore). The purity of the sample was verified by SDS-PAGE and silver staining. To remove the N-terminal 6xHis tag, Ku was incubated at 16°C overnight with AcTEV protease (Invitrogen) in buffer [50 mM Tris-HCl pH 8.0, 300 mM NaCl, 0.5 mM EDTA, 1 mM DTT]. The digested sample was diluted 10-fold with buffer (50 mM Tris pH 8 and 300 mM NaCl) and then loaded again onto a Ni-NTA column. The Ku containing fractions were combined and concentrated via a 50NMDL Amicon filter (Millipore) in 50 mM Tris-HCl pH 8.0, 300 mM NaCl. Storage buffer (0.5 mM EDTA, 1 mM DTT, 40% glycerol) was added and Ku was then aliquoted, flash frozen and stored at −80°C.

### Heterodimerization assays

YAB226 (*yku70-Δ yku80-Δ*) was co-transformed with FLAG-tagged versions of *YKU70, yku70-p1, yku70-R456E,* or *yku70-K422E* and myc-tagged versions of *YKU80* or *yku80-p1*. Fifty ml asynchronous cultures were lysed with glass beads in TMG-200 (10 mM Tris-HCl, pH 8.0, 1 mM MgCl_2_, 10% [v/v] glycerol, 0.1 mM DTT, 0.1 mM EDTA plus 200 mM NaCl). Forty µl of anti-FLAG agarose (Sigma A220) was added to 150 µg of protein extract. After 3 hours of incubation at 4°C, the beads were washed in TMG-200 plus 0.5% Tween and resuspended in 40 µl TMG. Input and IP samples were subjected to SDS-PAGE on 10% gels and analyzed by western blotting with anti-myc (9E10, Sigma M4439) and anti-PGK (Molecular Probes A6457) primary antibodies and IRDye 800CW conjugated goat anti-mouse secondary antibody (LiCor). Fluorescence was visualized using the LiCor Odyssey® Infared Imaging System. After quantitation was performed using ImageQuant software (Molecular Dynamics), the membrane was stripped and re-probed with anti-FLAG (M2, Sigma F3165).

### Yeast two-hybrid assays

YAB327 is a *yku70-Δ* derivative of EGY48 [20], which was grown on 5-FOA-containing media to select against the presence of the *URA3*-containing *LacZ* reporter plasmid. YAB327 was transformed with *YKU70* (pVL1874), *yku70-R456E* (pAB558), *yku70-p1* (pAB608), *yku70-K422E* (pAB710) or pRS416 and then a pEG202-based BAIT plasmid containing *YKU80* (pEGku80) or *yku80-p1* (pAB599) and a pJG4-5-based PREY vector containing full-length Sir4 (pB42AD::*SIR4*). Interaction between the BAIT and PREY constructs was determined by growth on Gal –Leu plates.

### TLC1 co-immunoprecipitation, RNA isolation, and quantitative RT-PCR

Procedures were performed using modified versions of described protocols [17], [54], [55]. Fifty mL cultures (A_600_ = 0.8–1.0) were lysed in TMG-50 using silica beads, and extracts containing approximately 4 mg protein were prepared. For samples immunoprecipitated with anti-myc antibody (9E10, Sigma M4439), Protein G beads (EMD Chemicals) were added after they were incubated overnight at 4°C. Both FLAG conjugated beads (Sigma A220) and Protein G beads were washed in TMG-50 plus 0.5% Tween before being resuspended in 50 µl TMG-50. The resuspended beads (and 50 µl aliquots of the inputs) were added to 350 çl of Proteinase K solution and incubated at 37°C for 30 minutes. Following phenol:chloroform extraction and ethanol precipitation, nucleic acids were resuspended in 100 µl RNase free water. The RNA was subjected to RNA cleanup and on-column DNase I treatment using the RNeasy kit (Qiagen). Two µg of input RNA (11 µl of IP RNA) was used as a template for cDNA synthesis using the Flex cDNA synthesis kit (Quanta Biosciences). Quantitative PCR was performed on 5 µl of the cDNA synthesis reaction using the Perfecta SYBR Green FastMix, ROX (Quanta Biosciences) on an ABI7300 real time PCR system (Applied Biosystems). Actin and TLC1 primers used were the same as described [54].

ABI7300 software was used to determine threshold and C_T_ values. C_T_ values were averaged and plotted using MS Excel. Percent input was determined by multiplying 100 by 2 ^(Adjusted input C^_T_^ – IP C^_T_^)^, where adjusted input C_T_  =  Input C_T_ - log_2_ (dilution factor).

### Chromatin immunoprecipitation (ChIP) assays

Fifty ml cultures of YTSF23 (*YKU70 YKU80*) [17], YTSF79 (*YKU70 YKU80_myc18_*) [17], YAB285 (*yku70-R456E YKU80_myc18_*), YAB438 (*yku70-p1 YKU80_myc18_*) and YAB439 (*yku70-p1 yku80-p1_myc18_*) were grown at 28°C to an A_600_ = 0.8–1.0. Formaldehyde crosslinking was carried out, as described by Aparicio [56]. Cell pellets were washed with 1 M sorbitol, resuspended in approximately 5 ml zymolyase buffer (1 M sorbitol, 50 mM Tris, 10 mM β-ME), and incubated with 125 µl zymolyase (50 mg/ml 20T zymolyase) for 1 hour at 28°C, as described [57]. Spheroplasts were pelleted and resuspended in approximately 500 µl NP-S buffer [57] before brief (2 minutes) glass bead lysis to further disrupt cells. The pellets were re-suspended in FA-lysis buffer [56] and sonicated with a Misonix Sonicator 3000 (power setting 5, 90 sec, 3 cycles). Total protein levels were adjusted so that equal amounts of protein were present in each sample. Five µl of 9E10 (Sigma) were added to IP samples and incubated overnight at 4°C. Protein G agarose beads (Calbiochem IP04) were equilibrated with lysis buffer, added to IP samples, and incubated at 4°C for 3 hours. Beads were washed at room temperature according to Aparacio, et al. [56]. Crosslinks were reversed, and the DNA was ethanol precipitated. The DNA fragments (∼500 bp) were re-suspended in 50 µl of TE and brought up to 300 µl with a 0.4N NaOH, 10 mM EDTA solution. The samples were heated to 100°C for 10 minutes before being blotted onto a Hybond XL nylon membrane (Amersham) using a dot blot manifold (Schleicher & Schuell). The blot was probed with a telomere-specific, end-labeled oligonucleotide (AB766) or a randomly labeled 3.2 kb DNA fragment containing TyB sequence excised from plasmid pAB126. After excess probe was washed away, the blot was exposed to phosphorimager screen for quantitation.

To monitor for efficiency of immunoprecipitation, 50 µl aliquots of the Input and IP samples were run on a 10% polyacrylamide gel, transferred to a nitrocellulose membrane, and probed with anti-myc (9E10, Sigma M4439) and anti-PGK (Molecular Probes A6457) primary antibodies and IRDye 800CW conjugated goat anti-mouse secondary antibody (LiCor).

### Telomere Southern blots and telomeric G-strand overhang assays

Southern blot analysis of telomeres and telomeric G-strand overhang assays were done as described previously [34], [58].

### Telomeric silencing assays

Plasmids containing WT or mutant *yku70* and *yku80* alleles or empty vector controls were co-transformed into YAB219 (*yku70-Δ yku80*-*Δ*) and YAB353 (*yku70-Δ yku80-Δ exo1-Δ*). Cultures were grown to mid-log phase in –Trp –Leu media. Five-fold serial dilutions were spotted onto –Trp –Leu, –Trp –Leu –Ura, and –Trp –Leu low ade plates, to monitor the expression of *URA3* and *ADE2*. Growth was determined after 2 to 4 days at 28°C. Temperature sensitivity was determined by plating the serial dilutions onto –Trp –Leu plates and incubating at 37°C for 2 to 4 days.

### Error-prone NHEJ assay

Plasmids containing *yku70* and/or *yku80* alleles or empty vector controls were transformed into YAB199 (*yku70-Δ*), YAB198 (*yku70-Δ*) or YAB273 (*yku70-Δ yku80-Δ*). Five-fold serial dilutions of asynchronous cultures were spotted onto –Trp and –Trp Gal plates (for YAB198 and YAB199) –Trp –Leu and –Trp –Leu Gal plates (YAB273). For single colony experiments, asynchronous cultures were diluted and 100 µl were spread onto these sets of plates. Colonies were counted after 2 to 4 days of incubation at 28°C.

### Statistical analyses

A student's T-test was used to determine whether the differences in values shown on bar graphs were statistically significant.

## Supporting Information

Figure S1 Purified recombinant WT and Yku70-R456E-containing Ku. One microgram of recombinant purified protein prior and after TEV-protease cleavage of the N terminal His_6_ tag. Proteins were resolved by 10% SDS-PAGE and visualized with Coomassie blue.
(TIF)

Figure S2 The *yku70-p1 yku80-p1* double mutant is markedly impaired for DNA end binding *in vitro*. To further examine the DNA end binding activity of the *yku70-p1 yku80-p1* double mutant, EMSA was performed with increasing amounts of WCE. The indicated amounts of WCE prepared from the designated strains were incubated with ^32^P end labeled 198 bp nontelomeric DNA fragment and 1000-fold excess of cold circular DNA and run on a nondenaturing polyacrylamide gel. Whereas increasing amounts of the ^32^P end labeled DNA fragment shifted with increasing amounts of WT WCE, a discrete shift was not observed in the *yku70-p1 yku80-p1* double mutant WCE, similar to the *yku80-Δ* WCE.
(TIF)

Figure S3 Chromatin immunoprecipitation of Yku70-p1/Yku80-p1 mutant Ku. Myc-tagged Yku80_ myc18_ or Yku80-p1_ myc18_ was immunoprecipitated from sheared chromatin prepared from crosslinked cells expressing Yku70, Yku70-R456E or Yku70-p1 as indicated. Isolated DNA was analyzed by dot-blot with a radiolabeled telomere specific probe or TyB probe. The mean percentage of telomeric DNA immunoprecipitated in three trials is shown +/- one SD. (B) Western blot showing Yku80_myc18_ or Yku80-p1_myc18_ immunoprecipitation efficiency in the designated samples. α-PGK serves as a control for protein levels for the input WCEs prior immunoprecipitation.
(TIF)

Figure S4 Telomere and imprecise NHEJ assays of *yku70-K422E.* (A) Telomeric silencing assay. Shown are five-fold serial dilutions of an *EXO1 yku70-Δ yku80-Δ* strain with VII-L*URA3* and V-R *ADE2* telomeric reporters (YAB219) transformed with plasmids containing vector, WT, or mutant versions of *YKU70* (*TRP1*) and *YKU80 (LEU2).* Growth was monitored on –Trp –Leu –Ura at 28°C to examine de-repression of *URA3* (-uracil plates), and on –Trp –Leu at 28°C to monitor plating efficiency (+uracil plates) and 37°C as a surrogate marker for telomere end protection (37°C plates) [26]. Growth was also examined on –Trp –Leu media with limiting Ade to examine de-repression of *ADE2* (low ade plates). (B) Telomeric length analysis. XhoI digested genomic DNA, isolated from a *yku70-Δ yku80-Δ* strain co-transformed with the indicated *CEN* plasmids (+,*YKU70* for top row or *YKU80*, for bottom row; *Δ*, empty vector; R, *yku70-R456E; p1, yku70-p1* for top row or *yku80-p1* for bottom row; -8, *yku80-8*; or K, *yku70-K422E*), was blotted and probed with an end labeled telomere specific oligo. (C) Quantitative imprecise NHEJ assay. The strains used [YAB198 (*yku70-Δ*) containing *YKU70*, *yku70-K422E,* or vector (*TRP1*)] contain an HO endonuclease under the control of a galactose-inducible promoter and a single HO cleavage site repairable by NHEJ only. The strains were plated on –Trp media containing galactose and –Trp media to determine colony survival under galactose-induced constitutive HO expression. Averages of 3 independent experiments are shown with error bars showing one SD.
(TIF)

Figure S5 The *yku80-8* strain demonstrates normal DNA end binding activity in the WCE EMSA. WCEs prepared from cells with indicated genotypes were incubated with 1000-fold excess cold circular DNA and a ^32^P end-labeled nontelomeric 198 bp DNA fragment and run on a nondenaturing polyacrylamide gel. The strains were created by transformation of *yku80-Δ* strain (YVL885) with a *YKU80,* empty vector, or *yku80-8* CEN plasmid.
(TIF)

Figure S6 An exo1-*Δ* suppresses the increased telomeric G-overhang in the DNA end binding defective Ku mutants. (A) Telomeric G-overhang assay. XhoI digested genomic DNA fragments isolated from *yku70-Δ yku80-Δ* strains transformed with *CEN* plasmids containing the indicated alleles (+, WT; *Δ*, empty vector; R, *yku70-R456E; p1, yku70-p1* for top row or *yku80-p1* for bottom row) of *YKU70* and *YKU80* were incubated with a radiolabeled telomere-specific oligomeric probe and run on a native gel (top). The amount of single-stranded telomeric DNA was quantitated before the gel was denatured and probed with the same telomere probe to reveal the total amount of telomeric DNA (bottom). (B) Ratio of single-stranded telomeric DNA to total telomeric DNA, normalized to WT. Values represent the average of three independent experiments. Error bars indicate one SD.
(TIF)

Table S1 Yeast strains used in this study.
(DOC)

Table S2 Plasmids used in this study.
(DOC)
