# Supplementary figures and images for: Ku Must Load Directly onto the Chromosome End in Order to Mediate Its Telomeric Functions
Source: PLoS Genet. 2011 Aug 11;7(8):e1002233. doi: 10.1371/journal.pgen.1002233 (PMC3154960; doi:10.1371/journal.pgen.1002233)

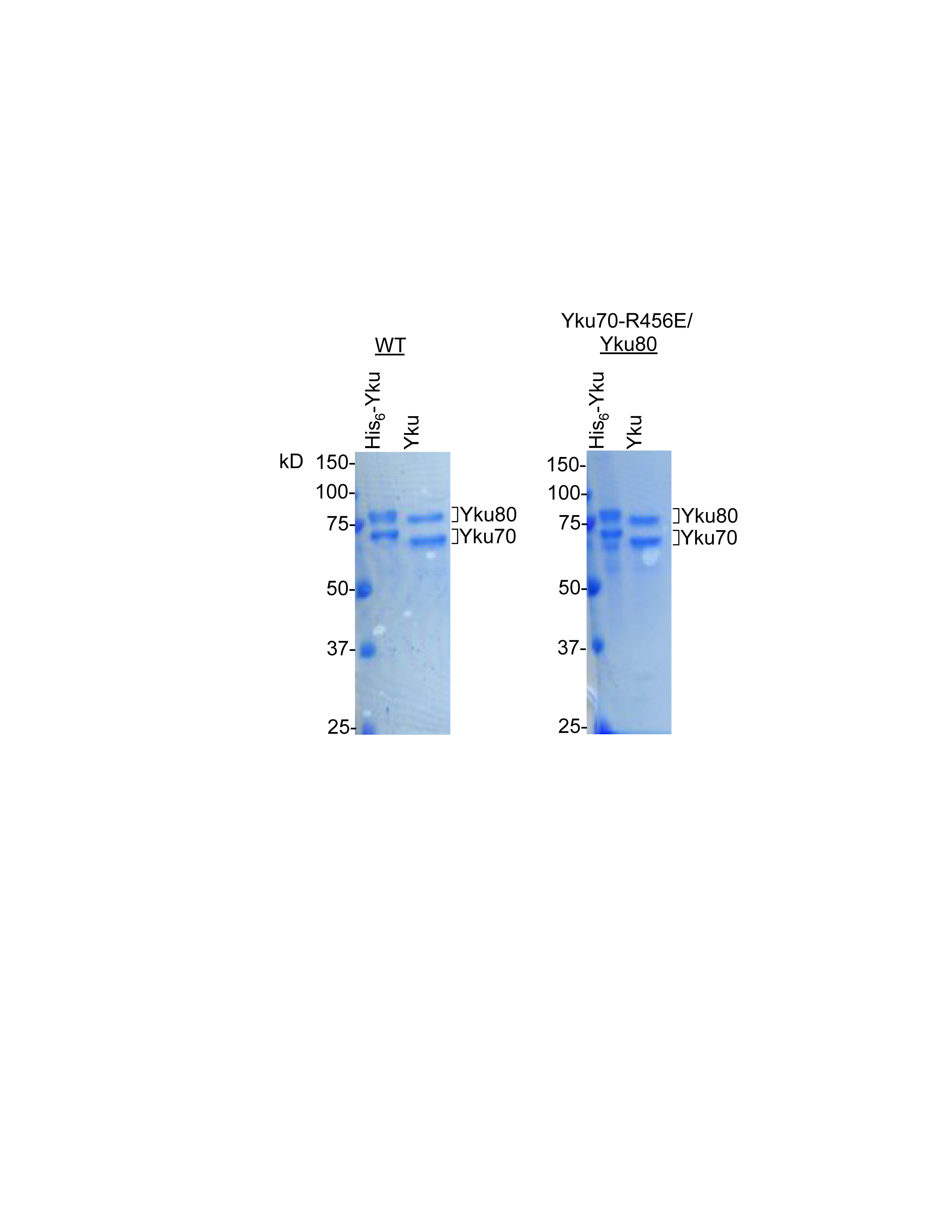

Supplement: Figure S1 — Purified recombinant WT and Yku70-R456E-containing Ku. One microgram of recombinant purified protein prior and after TEV-protease cleavage of the N terminal His6 tag. Proteins were resolved by 10% SDS-PAGE and visualized with Coomassie blue. (TIF) [file pgen.1002233.s001.tif]

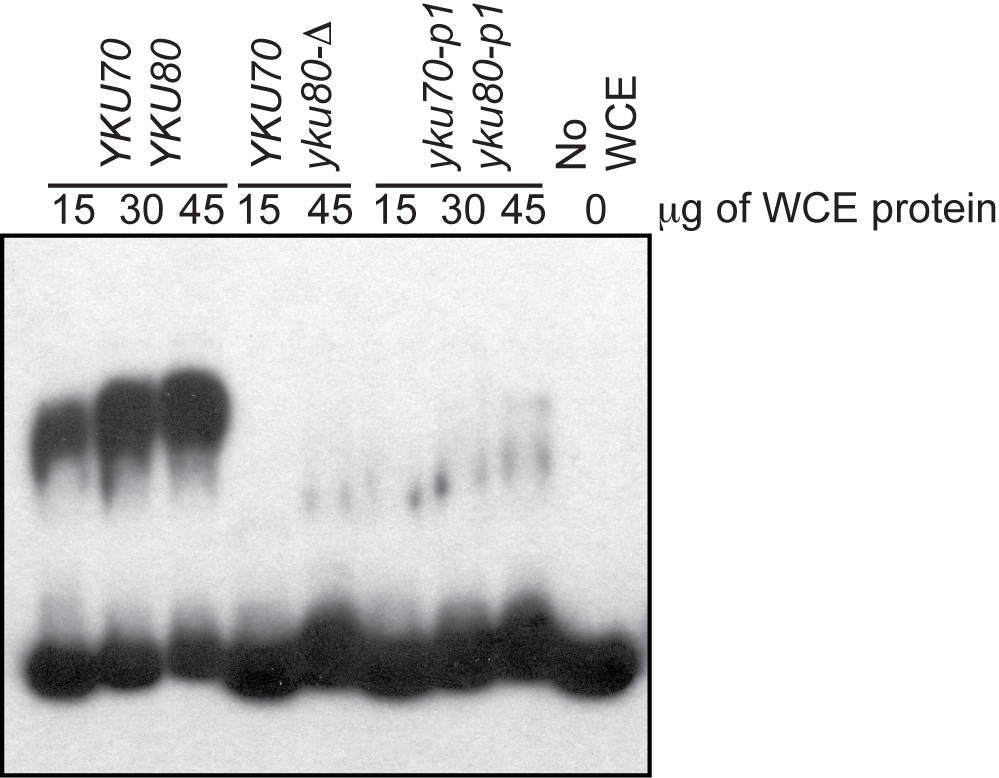

Supplement: Figure S2 — The yku70-p1 yku80-p1 double mutant is markedly impaired for DNA end binding in vitro. To further examine the DNA end binding activity of the yku70-p1 yku80-p1 double mutant, EMSA was performed with increasing amounts of WCE. The indicated amounts of WCE prepared from the designated strains were incubated with 32P end labeled 198 bp nontelomeric DNA fragment and 1000-fold excess of cold circular DNA and run on a nondenaturing polyacrylamide gel. Whereas increasing amounts of the 32P end labeled DNA fragment shifted with increasing amounts of WT WCE, a discrete shift was not observed in the yku70-p1 yku80-p1 double mutant WCE, similar to the yku80-Δ WCE. (TIF) [file pgen.1002233.s002.tif]

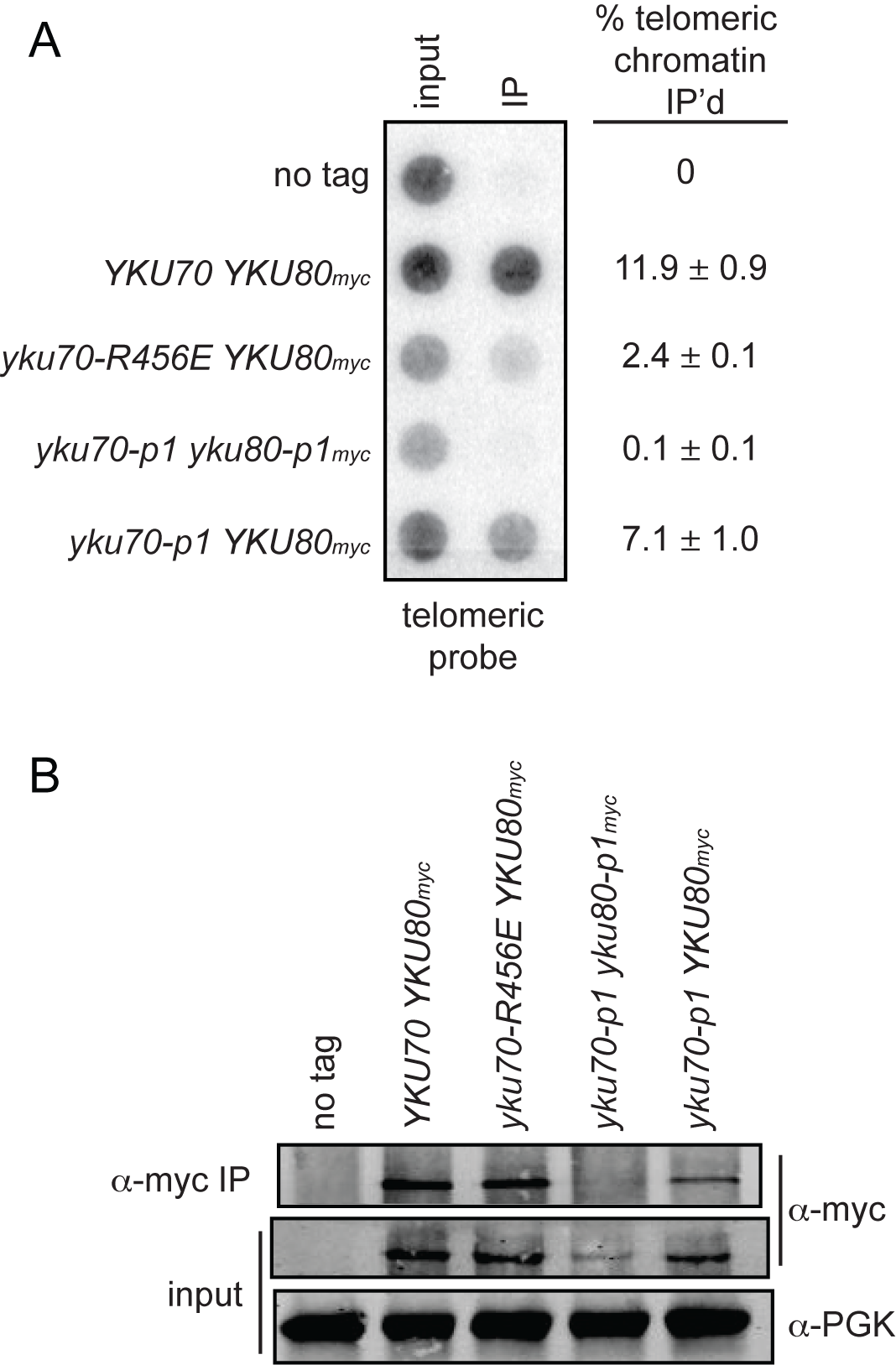

Supplement: Figure S3 — Chromatin immunoprecipitation of Yku70-p1/Yku80-p1 mutant Ku. Myc-tagged Yku80 myc18 or Yku80-p1 myc18 was immunoprecipitated from sheared chromatin prepared from crosslinked cells expressing Yku70, Yku70-R456E or Yku70-p1 as indicated. Isolated DNA was analyzed by dot-blot with a radiolabeled telomere specific probe or TyB probe. The mean percentage of telomeric DNA immunoprecipitated in three trials is shown +/- one SD. (B) Western blot showing Yku80myc18 or Yku80-p1myc18 immunoprecipitation efficiency in the designated samples. α-PGK serves as a control for protein levels for the input WCEs prior immunoprecipitation. (TIF) [file pgen.1002233.s003.tif]

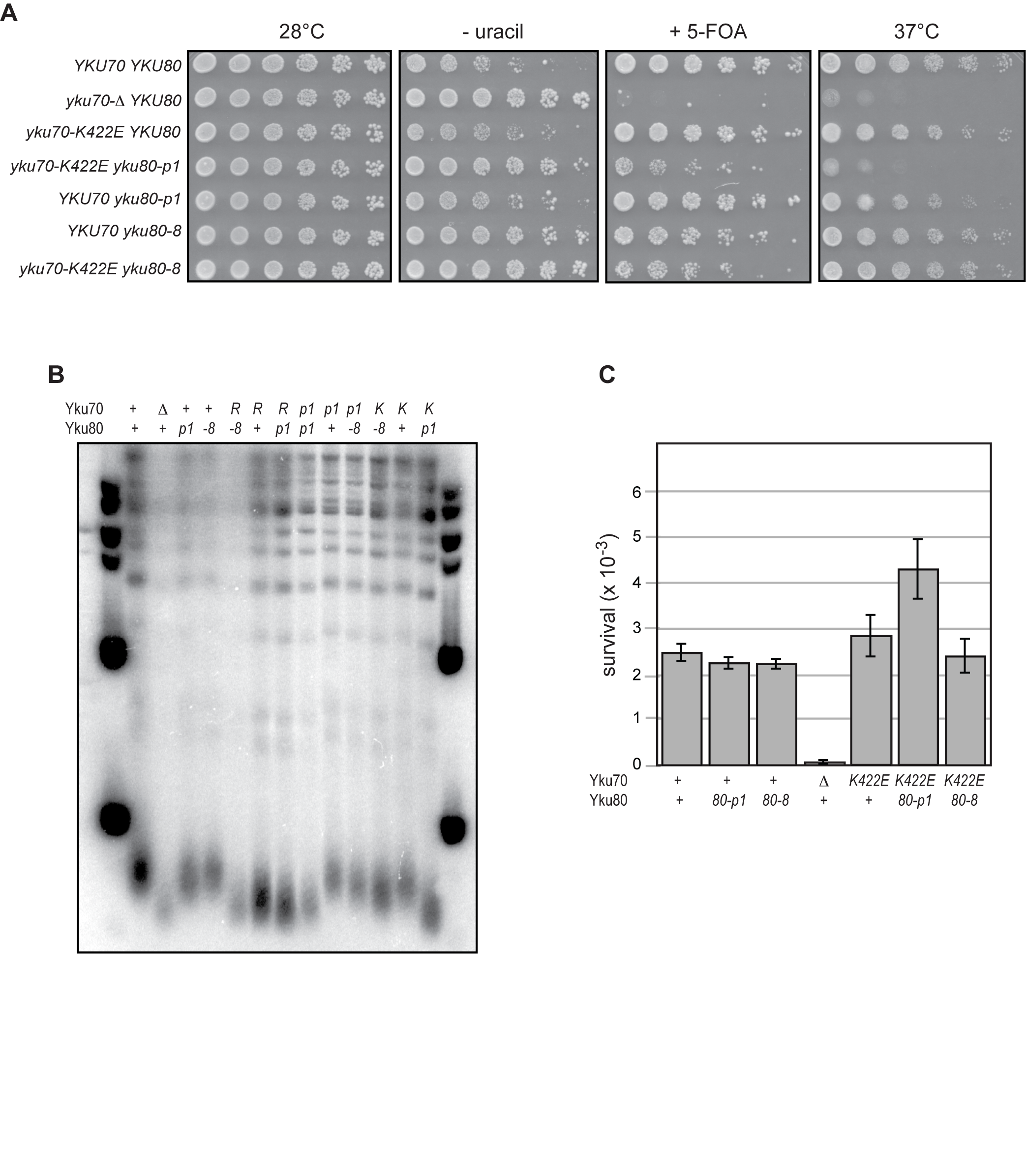

Supplement: Figure S4 — Telomere and imprecise NHEJ assays of yku70-K422E. (A) Telomeric silencing assay. Shown are five-fold serial dilutions of an EXO1 yku70-Δ yku80-Δ strain with VII-LURA3 and V-R ADE2 telomeric reporters (YAB219) transformed with plasmids containing vector, WT, or mutant versions of YKU70 (TRP1) and YKU80 (LEU2). Growth was monitored on –Trp –Leu –Ura at 28°C to examine de-repression of URA3 (-uracil plates), and on –Trp –Leu at 28°C to monitor plating efficiency (+uracil plates) and 37°C as a surrogate marker for telomere end protection (37°C plates) [26]. Growth was also examined on –Trp –Leu media with limiting Ade to examine de-repression of ADE2 (low ade plates). (B) Telomeric length analysis. XhoI digested genomic DNA, isolated from a yku70-Δ yku80-Δ strain co-transformed with the indicated CEN plasmids (+,YKU70 for top row or YKU80, for bottom row; Δ, empty vector; R, yku70-R456E; p1, yku70-p1 for top row or yku80-p1 for bottom row; -8, yku80-8; or K, yku70-K422E), was blotted and probed with an end labeled telomere specific oligo. (C) Quantitative imprecise NHEJ assay. The strains used [YAB198 (yku70-Δ) containing YKU70, yku70-K422E, or vector (TRP1)] contain an HO endonuclease under the control of a galactose-inducible promoter and a single HO cleavage site repairable by NHEJ only. The strains were plated on –Trp media containing galactose and –Trp media to determine colony survival under galactose-induced constitutive HO expression. Averages of 3 independent experiments are shown with error bars showing one SD. (TIF) [file pgen.1002233.s004.tif]

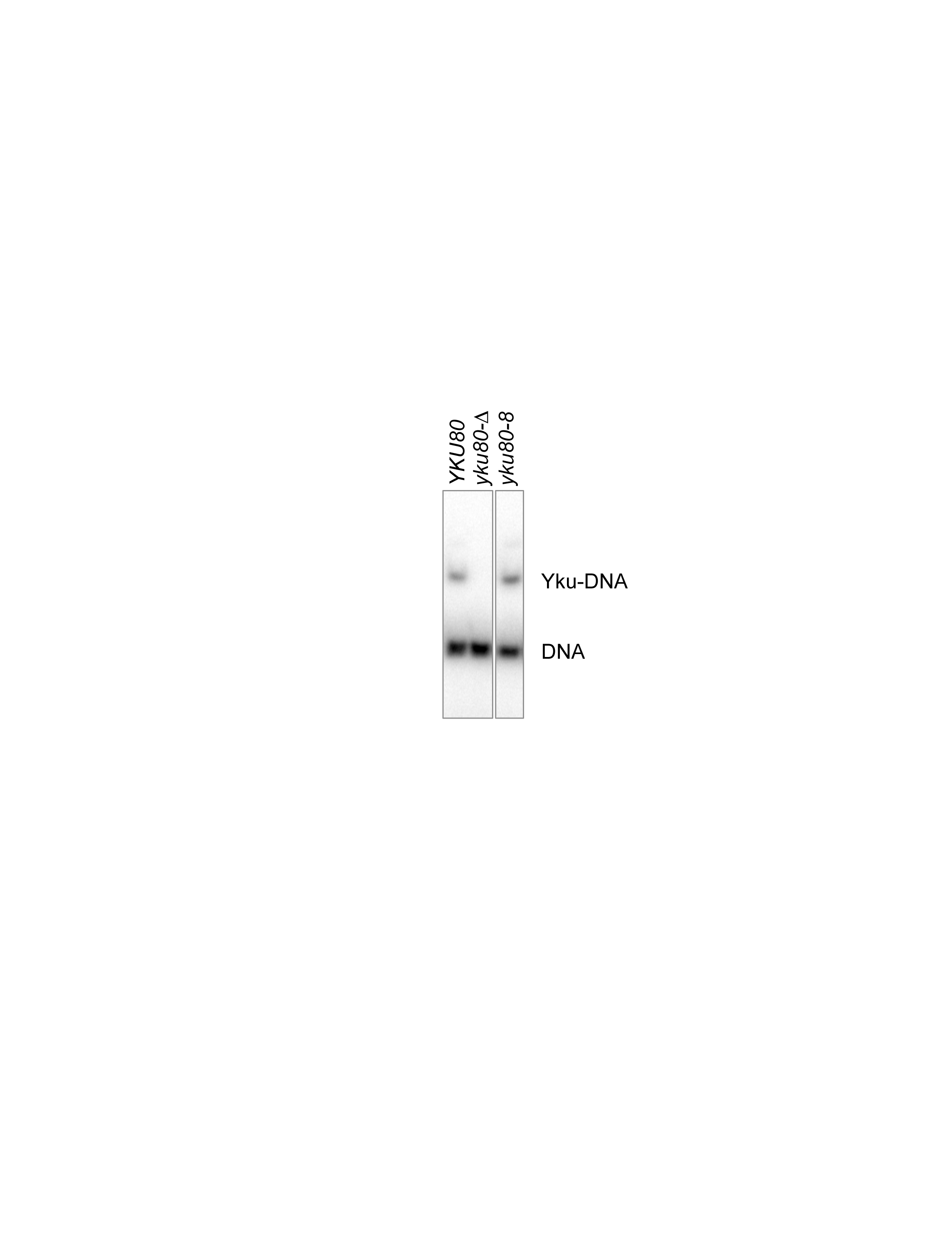

Supplement: Figure S5 — The yku80-8 strain demonstrates normal DNA end binding activity in the WCE EMSA. WCEs prepared from cells with indicated genotypes were incubated with 1000-fold excess cold circular DNA and a 32P end-labeled nontelomeric 198 bp DNA fragment and run on a nondenaturing polyacrylamide gel. The strains were created by transformation of yku80-Δ strain (YVL885) with a YKU80, empty vector, or yku80-8 CEN plasmid. (TIF) [file pgen.1002233.s005.tif]

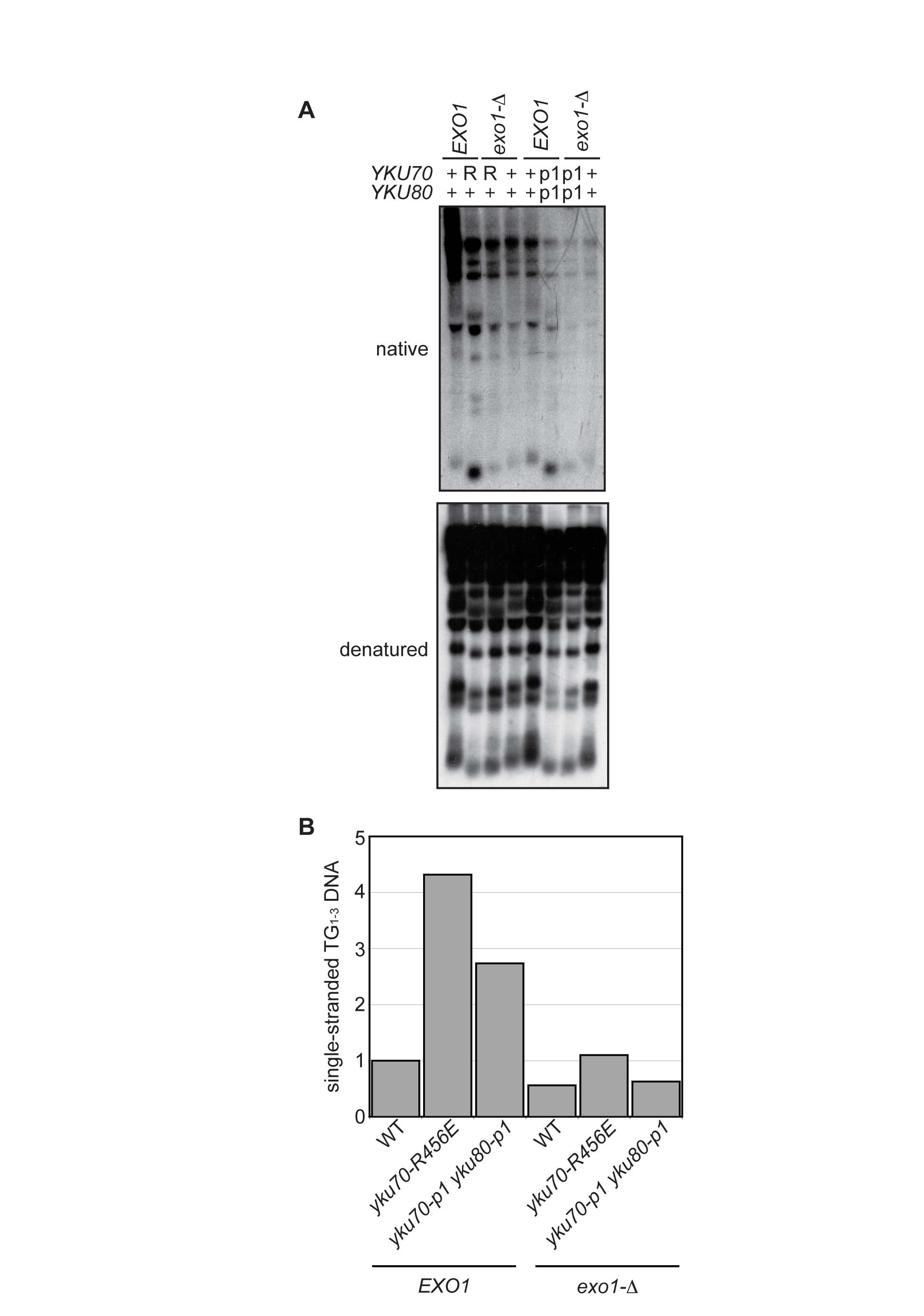

Supplement: Figure S6 — An exo1-Δ suppresses the increased telomeric G-overhang in the DNA end binding defective Ku mutants. (A) Telomeric G-overhang assay. XhoI digested genomic DNA fragments isolated from yku70-Δ yku80-Δ strains transformed with CEN plasmids containing the indicated alleles (+, WT; Δ, empty vector; R, yku70-R456E; p1, yku70-p1 for top row or yku80-p1 for bottom row) of YKU70 and YKU80 were incubated with a radiolabeled telomere-specific oligomeric probe and run on a native gel (top). The amount of single-stranded telomeric DNA was quantitated before the gel was denatured and probed with the same telomere probe to reveal the total amount of telomeric DNA (bottom). (B) Ratio of single-stranded telomeric DNA to total telomeric DNA, normalized to WT. Values represent the average of three independent experiments. Error bars indicate one SD. (TIF) [file pgen.1002233.s006.tif]
