# Supplementary material for: Ku Must Load Directly onto the Chromosome End in Order to Mediate Its Telomeric Functions
Source: PLoS Genet. 2011 Aug 11;7(8):e1002233. doi: 10.1371/journal.pgen.1002233 (PMC3154960; doi:10.1371/journal.pgen.1002233)
Supplement: Table S1 — Yeast strains used in this study. (DOC) [file pgen.1002233.s007.doc]

**Table S1. Yeast Strains**

**Strain Genotype**

DRY2805 *MAT***a** *trp1-901 leu2-3,112 ura3-52 his3-200 gal4-∆ gal80-∆ LYS::GAL-HIS3 GAL2-ADE2 met::GAL7-lacZ*

YVL885  *MAT***a** *yku80-∆::kan^r^ ura3-52 lys2-801 ade2-101 trp1-∆63 his3-∆200 leu2-∆1 ppr1::HIS3 adh4::URA3-(URA3* at TEL VIIL*) DIA5-1 (ADE2* at TEL VR*)*

YAB198 *MAT***α** *yku70-∆::kan^r^ hml-∆::ADE1 hmr-∆::ADE1 ade1-110 leu2,3-112 lys5 trp1-∆::hisG ura3-52 ade3::GAL10 HO*

YAB199 *MAT***α** *yku70-∆::kan^r^ tlc1∆::LEU2 hml-∆::ADE1 hmr-∆::ADE1 ade1-110 leu2,3-112 lys5 trp1-∆::hisG ura3-52 ade3::GAL10 HO* pSD120 (*CEN URA3 TLC1*)

YAB219 *MAT***a** *yku70-∆::nat^r^ yku80-∆::kan^r^ ura3-52 lys2-801 ade2-101 trp1-∆63 his3-∆200 leu2-∆1 ppr1-∆::HIS3 adh4::URA3-(URA3* at TEL VIIL*)* DIA5-1 (*ADE2* at TEL VR)

YAB226 *MAT***a** *yku70-∆::nat^r^ yku80-∆::kan^r^* *leu2 trp1 ura3-52 GAL+ prb- prc- pep4-3*

YAB273 *MAT***a** *yku70-∆::kan^r^ yku80-∆::kan^r^ hml-∆::ADE1 hmr-∆::ADE1 ade1-110 leu2,3-112 lys5 trp1-∆::hisG ura3-52 ade3::GAL10 HO*

YAB285 *MAT***a** *yku70-R456E* *YKU80-G8_myc18_::TRP1 TEL VII-L::URA3 bar1-Δ::kan^r^ ura3-52 lys2-801_amber ade2-101_ochre trp1-Δ63 his3-Δ200 leu2-Δ1*

YAB327 *MAT***α** *yku70-∆::kan^r^ ura3 his3 LEU::pLexAop6-LEU2*

YAB353 *MAT***a** *yku70-∆*::*nat^r^* *yku80-∆::kan^r^ exo1-∆::hygro^r^* *ura3-52 lys2-801 ade2-101 trp1-∆63 his3-∆200 leu2-∆1 ppr1-∆::HIS3 adh4::URA3-*TEL DIA5-1 (*ADE2* at TEL VR)

YAB438 *MAT***a** *yku70-p1* *YKU80-G8_myc18_::TRP1 TEL VII-L::URA3 bar1Δ::kan^r^ ura3-52 lys2-801_amber ade2-101_ochre trp1-Δ63 his3-Δ200 leu2-Δ1*

YAB439 *MAT***a** *yku70-p1* *yku80-p1-G8_myc18_::TRP1 TEL VII-L::URA3 bar1-Δ::kan^r^ ura3-52 lys2-801_amber ade2-101_ochre trp1-Δ63 his3-Δ200 leu2-Δ1*

YTSF23 *MAT***a** *TEL VII-L::URA, bar1-Δ::kan^r^ ura3-52 lys2-801_amber ade2-101_ochre trp1-Δ63 his3-Δ200 leu2-Δ1*

YTSF79 *MAT***a** *YKU80-G8_myc18_::TRP1 TEL VII-L::URA3 bar1-Δ::kan^r^ ura3-52 lys2-801_amber ade2-101_ochre trp1-Δ63 his3-Δ200 leu2-Δ1*

YTSF98 *MAT***a** *tlc1-Δ48 YKU80-G8_myc18_::TRP1 TEL VII-L::URA3 bar1-Δ::kan^r^ ura3-52 lys2-801_amber ade2-101_ochre trp1-Δ63 his3-Δ200 leu2-Δ1*

**References**

1. Vandre CL, Kamakaka RT, Rivier DH (2008) The DNA end-binding protein Ku regulates silencing at the internal HML and HMR loci in Saccharomyces cerevisiae. Genetics 180: 1407-1418.

2. Bertuch AA, Lundblad V (2003) The Ku heterodimer performs separable activities at double strand breaks and chromosome termini. Mol Cell Biol 23: 8202-8215.

3. Ribes-Zamora A, Mihalek I, Lichtarge O, Bertuch AA (2007) Distinct faces of the Ku heterodimer mediate DNA repair and telomeric functions. Nat Struct Mol Biol 14: 301-307.

4. Fisher TS, Taggart AK, Zakian VA (2004) Cell cycle-dependent regulation of yeast telomerase by Ku. Nat Struct Mol Biol 11: 1198-1205.
