# Supplementary material for: Ku Must Load Directly onto the Chromosome End in Order to Mediate Its Telomeric Functions
Source: PLoS Genet. 2011 Aug 11;7(8):e1002233. doi: 10.1371/journal.pgen.1002233 (PMC3154960; doi:10.1371/journal.pgen.1002233)
Supplement: Table S2 — Plasmids used in this study. (DOC) [file pgen.1002233.s008.doc]

Table S2. Plasmids

| **Plasmid** | **Description** |
| --- | --- |

pAB178 *CEN TRP1 yku80-R401W*

pAB179 *CEN TRP1 yku80-N407D*

pAB180 *CEN TRP1 yku80-R443E*

pAB197 *CEN TRP1 yku70-R265E*

pAB198 *CEN TRP1 yku70-R456E*

pAB199 *CEN TRP1 yku70-K422E*

pAB279 *CEN TRP1 yku70-IMFQ266-269QDEY*

pAB348 *CEN LEU2 yku80-SKKDS400-404DEEDD*

pAB508 *LYS2 yku70-R456E*

pAB531 *CEN TRP1 YKU70_3xFLAG_*

pAB537 *CEN TRP1 yku70-R456E_3xFLAG_*

pAB538 *CEN TRP1 yku70-IMFQ266-269QDEY_3xFLAG_*

pAB546 *CEN LEU2 yku80-SKKDS400-404DEEDD_3xFLAG_*

pAB548 *CEN LEU2 YKU80_3xFLAG_*

pAB549 *CEN URA3 yku80-SKKDS400-404DEEDD_myc_*

pAB558 *CEN URA3 yku70-R456E*

pAB599 *HIS3 yku80-SKKDS400-404DEEDD*

pAB606 2µ *TRP1 yku70-R456E*

pAB607 *2*µ *TRP1 yku70-IMFQ266-269QDEY*

pAB608 *CEN URA3 yku70-IMFQ266-269QDEY*

pAB696 *CEN TRP1 yku70-K422E_3xFLAG_*

pAB710 *CEN URA3 yku70-K422E*

pB42AD::*SIR4*  2µ *TRP1 B42AD::SIR4*

pEG-KU80 2µ *HIS3 LexA::YKU80*

pEG202 2µ *HIS3 LexA*

pJG4-5 2µ *TRP1 B42AD*

pRS414 *CEN TRP1*

pRS415 *CEN LEU2*

pRS416 *CEN URA3*

pVL1057 *CEN TRP1 YKU70*

pVL1067 *CEN LEU2 YKU80*

pVL1069 *CEN URA3 YKU80*

pVL1352 *CEN URA3 YKU80_myc_*

pVL1874 *CEN URA3 YKU70*

**References**

1. Roy R, Meier B, McAinsh AD, Feldmann HM, Jackson SP (2004) Separation-of-function mutants of yeast Ku80 reveal a Yku80p-Sir4p interaction involved in telomeric silencing. J Biol Chem 279: 86-94.

2. Christianson TW, Sikorski RS, Dante M, Shero JH, Hieter P (1992) Multifunctional yeast high-copy-number shuttle vectors. Gene 110: 119-122.

3. Ribes-Zamora A, Mihalek I, Lichtarge O, Bertuch AA (2007) Distinct faces of the Ku heterodimer mediate DNA repair and telomeric functions. Nat Struct Mol Biol 14: 301-307.

4. Bertuch AA, Lundblad V (2003) The Ku heterodimer performs separable activities at double strand breaks and chromosome termini. Mol Cell Biol 23: 8202-8215.
